# Inhibiting bacterial cooperation is an evolutionarily robust anti-biofilm strategy

**DOI:** 10.1038/s41467-019-13660-x

**Published:** 2020-01-09

**Authors:** Lise Dieltjens, Kenny Appermans, Maries Lissens, Bram Lories, Wook Kim, Erik V. Van der Eycken, Kevin R. Foster, Hans P. Steenackers

**Affiliations:** 1 Department of Microbial and Molecular Systems, Centre of Microbial and Plant Genetics (CMPG), KU Leuven, Leuven, Belgium; 2 Department of Zoology and Department of Biochemistry, University of Oxford, Oxford, UK; 3 Department of Biological Sciences, Duquesne University, Pittsburgh, USA; 4 Department of Chemistry, Laboratory for Organic & Microwave-Assisted Chemistry (LOMAC), KU Leuven, Leuven, Belgium; 5 Peoples’ Friendship University of Russia (RUDN University), 6 Miklukho-Maklaya street, Moscow, Russia

**Keywords:** Antimicrobial resistance, Bacteriology, Biofilms, Microbial communities

## Abstract

Bacteria commonly form dense biofilms encased in extracellular polymeric substances (EPS). Biofilms are often extremely tolerant to antimicrobials but their reliance on shared EPS may also be a weakness as social evolution theory predicts that inhibiting shared traits can select against resistance. Here we show that EPS of *Salmonella* biofilms is a cooperative trait whose benefit is shared among cells, and that EPS inhibition reduces both cell attachment and antimicrobial tolerance. We then compare an EPS inhibitor to conventional antimicrobials in an evolutionary experiment. While resistance against conventional antimicrobials rapidly evolves, we see no evolution of resistance to EPS inhibition. We further show that a resistant strain is outcompeted by a susceptible strain under EPS inhibitor treatment, explaining why resistance does not evolve. Our work suggests that targeting cooperative traits is a viable solution to the problem of antimicrobial resistance.

## Abstract (web summary)

Bacterial biofilms rely on shared extracellular polymeric substances (EPS) and are often highly tolerant to antibiotics. Here, the authors show in in vitro experiments that *Salmonella* does not evolve resistance to EPS inhibition because such strains are outcompeted by a susceptible strain under inhibitor treatment.

## Introduction

Biofilms are a major form of microbial life in which bacteria form dense surface-associated communities, typically enclosed in a matrix of self-produced exopolymeric substances (EPS)^1–3^. Bacteria within biofilms are up to 1,000 times more tolerant to antibiotics, disinfectants, mechanical removal, and other stresses, and this tolerance heavily impedes antimicrobial treatment^4,5^. Hence, persistent biofilm infections and contaminations widely occur and cause a tremendous amount of problems in various sectors, including medicine^6,7^, food industry^8,9^ and agriculture^9,10^. This urges the need for strategies that inhibit biofilm formation and render microbes susceptible to treatment. Several approaches have been proposed ranging from blocking bacterial attachment, to inhibiting or destabilizing EPS, and interfering with quorum sensing^11–13^. Given the limited permeability of established biofilms, particular promise comes from strategies that continually treat surfaces to prevent the formation of biofilms^14^.

As for antibiotics^15,16^, the problem with any long-term treatment strategies is the potential for resistance evolution. We need anti-biofilm strategies, therefore, that also limit the evolution of resistance. One option is to use combinations of therapies, which use multiple drugs to limit resistance^17^. More desirable still would be the discovery of single strategies where resistance evolution never occurs, so called evolution proof strategies^18^. The notion of an evolution proof antimicrobial strategy is often considered an unrealistic prospect because eventually, a mutation or mutations rendering a strain resistant appears inevitable^19^. However, an ingenious solution to this problem has been proposed^20^. This accepts the inevitability of resistant variants but focuses on making sure they are not favoured by natural selection. The idea rests on inhibiting the social traits of bacteria^21,22^. More specifically, it focuses on what are often known as public goods, secreted products that are costly to produce but benefit other cells in the population^23^. Examples are the secretion of enzymes that break down complex molecules for import^24^ or polymers that help cells to bind to a surface and one another^25^.

Social evolution theory predicts that inhibiting public goods inhibits bacterial growth and survival^23,26^, and that, critically, strains resistant to the public good inhibitor will be counter selected. The reason is that a resistant strain will produce the public good and pay a cost to do so, while susceptible strains will be able to use the public good without paying the cost^18,27,28^. In support of this, work in *Pseudomonas aeruginosa*, in the absence of a drug, showed that public goods can be exploited by non-producers at low producer frequencies, suggesting that resistance would be counter-selected^21,22^. Furthermore, evolution experiments in *P. aeruginosa* indicated that inhibiting public pyoverdine siderophores by gallium is evolutionarily robust, although it was not directly demonstrated that resistant strains were counter selected^29,30^.

We hypothesised that biofilms are an ideal target to develop and test the idea of public goods inhibition. The high cell density and secreted substances that make biofilms so resilient, also make them particularly reliant on public goods^31,32^. This suggests that biofilms may be particularly susceptible to strategies targeting resistance evolution. We, therefore, sought to design a treatment strategy that inhibited biofilm and, critically, where we can demonstrate that resistant strains are counter selected. Our approach centers upon in-house developed 5-aryl-2-aminoimidazole-based inhibitors^14,33–36^ of EPS production in *Salmonella* biofilms. *Salmonella* species form biofilms outside and inside the host and EPS both helps cells attach and protects against eradication by mechanical cleaning, disinfectants, antibiotics and the host immune system^37^. Inhibiting EPS, therefore, offers a route to inhibit biofilms, and reduce major problems and economic losses due to *Salmonella* in industrial^9^ and medical settings^38^. Moreover, EPS has the potential to be shared with other cells, rendering it a potential public good^39^, meaning that its inhibition may be subject to the hypothesized counter-selection of resistance.

Below, we demonstrate that EPS is indeed a public good in *Salmonella* biofilms, that resistance does not evolve under 40 days of EPS inhibitor treatment and, most importantly, we find a resistant strain and demonstrate that this is outcompeted by a susceptible strain when we treat biofilms with the inhibitor. Our work suggests that public good inhibition is effective against biofilms, and, more generally, as a way to combat the rise of antimicrobial resistance.

## Results and discussion

### *Salmonella* EPS is a public good

Biofilms play a crucial role in the survival of the food-borne pathogen *Salmonella* *Typhimurium*, outside (in food industry and agriculture)^37^ as well as inside the host (colonization of gallstones;^40,41^ protection against phagocytes;^42^ a role in gut colonization remains elusive^43,44^). The main EPS components are curli fimbriae^45^ and cellulose^46^, which form a dense interwoven network controlled by the master regulator of EPS, CsgD^47^. The production of curli is stimulated by CsgD by transcriptional activation of the *csgBAC* operon^48^. Cellulose production occurs via transcriptional activation of the diguanylate cyclase AdrA^46^, which in turn produces the second messenger c-di-GMP that relieves auto-inhibition of the cellulose synthase BcsAB^49^. To show that *Salmonella* EPS is a public good that is suitable for our strategy we have to demonstrate that (i) EPS increases biofilm formation and antimicrobial tolerance, (ii) EPS is costly to producing cells and (iii) EPS made by one cell benefits other cells.

**Fig. 1:**
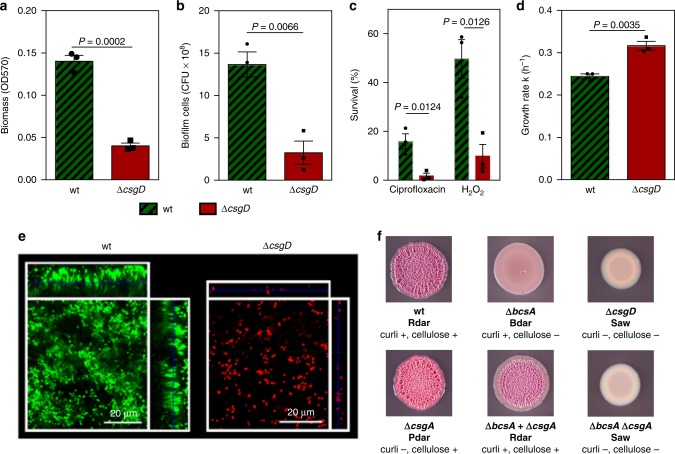
Salmonella biofilm EPS is a public good. Wild-type *S*. *Typhimurium* strain ATCC14028 (EPS producer) is indicated in (shaded) green; the isogenic Δ*csgD* mutant (EPS non-producer) is indicated in red. **a** Amount of biomass in monoculture biofilms. **b** Number of cells in monoculture biofilms. **c** Survival of monoculture biofilms after treatment with ciprofloxacin (1 µm) and H_2_O_2_ (0.25%). **d** Average growth rate (∆CFU h^−1^) in competition (1:1) during late exponential phase in liquid (8–20 h). The relative growth rate of wild type compared with Δ*csgD* mutant is 1.3. **e** Confocal image of monoculture biofilms. **f** Colony morphologies of the wild type and different biofilm mutants. For competition experiments **d** and microscopy **e**, wild type and Δ*csgD* mutant were fluorescently labelled in green and red, respectively. A similar outcome was obtained when colors were reversed and competition was neutral when strains that only differ in the fluorescent protein marker were competed against each other (Supplementary Fig. 2). Bars represent mean, dots represent measurements for biological replicates and error bars show s.e.m. (*n* = 3 biologically independent samples). *P* values derived from two-tailed student’s *t* test using Welch’s correction if s.d. are significantly (*P* < 0.05) different. Source data are provided as a Source Data file.

To investigate whether EPS production enhances the amount and antimicrobial tolerance of the biofilm, we grew the *S*. *Typhimurium* ATCC14028 wild type strain and an isogenic Δ*csgD* mutant for 48 h in monoculture biofilms on the bottom of polystyrene petridishes, filled with nutrient-poor liquid broth. These growth conditions are comparable to a multitude of situations where *Salmonella* biofilms form and cause problems in industrial settings^37^. As indicated in Fig. 1a, b, the Δ*csgD* mutant, which makes little EPS^50,51^, shows a strongly reduced biofilm formation compared to the wild type, with cell numbers decreased by 76% and biomass down 72% (as measured by crystal violet staining that mostly targets EPS). Confocal microscopy shows that the Δ*csgD* mutant forms a thin, unstructured layer of cells (±20 µm) while the wild type biofilm is thicker and shows cell clusters (±60 µm) (Fig. 1e). Moreover, the EPS-deficient Δ*csgD* mutant is much more sensitive to hydrogen peroxide, a commonly used disinfectant in food industry^43^, and ciprofloxacin, an antibiotic commonly used to treat *Salmonella* infections^52^ (Fig. 1c). Consistently, a cellulose-deficient mutant in *S*. Enteritidis was previously reported to be more sensitive to chlorine^53^. Overall, these results indicate that EPS production enhances biofilm functioning by both increasing cell numbers and tolerance to antimicrobials, which supports the idea of EPS inhibitors as an anti-biofilm strategy.

We next asked whether EPS production is costly by competing wild type *S*. *Typhimurium* with the isogenic Δ*csgD* mutant in well-mixed liquid cultures in test tubes. *csgD* is expressed during late-exponential phase^50,54^. The relative growth rate of the Δ*csgD* mutant in comparison with the wild type during this period (8–20 h p.i.) is 1.3, pointing to a significant fitness cost for EPS production (Fig. 1d). A significant growth cost was also seen during biofilm formation in nutrient-filled petridishes, with the Δ*csgD* mutant achieving higher total cellular yields (sum of biofilm and planktonic cells) than the wild type (Supplementary Fig. 1).

Finally, we investigated whether the EPS of one cell benefits other cells. To do this, we first asked whether EPS is shared between a producer and non-producer strain. Wild-type *S*. *Typhimurium* colonies, expressing both the extracellular matrix components curli and cellulose, show a rdar (red dry and rough) morphotype on Congo Red (CR)-agar plates, whereas a Δ*csgD* mutant shows a saw (smooth and white) morphotype^44^ (Fig. 1f). A Δ*csgA* mutant, lacking the major curlin subunit CsgA, shows a pdar (pink dark and rough) morphotype, whereas a Δ*bcsA* mutant, lacking the cellulose synthase catalytic subunit BcsA, shows a bdar (brown dark and rough) morphotype. Colonies formed by a mixture of the Δ*csgA* mutant and Δ*bcsA* mutant restore the wild-type rdar morphotype, suggesting that the EPS components are shared between cells (Fig. 1f). These data are consistent with previous (rdar) colony studies in *Salmonella*^54^ and in *Bacillus*^55^.

**Fig. 2:**
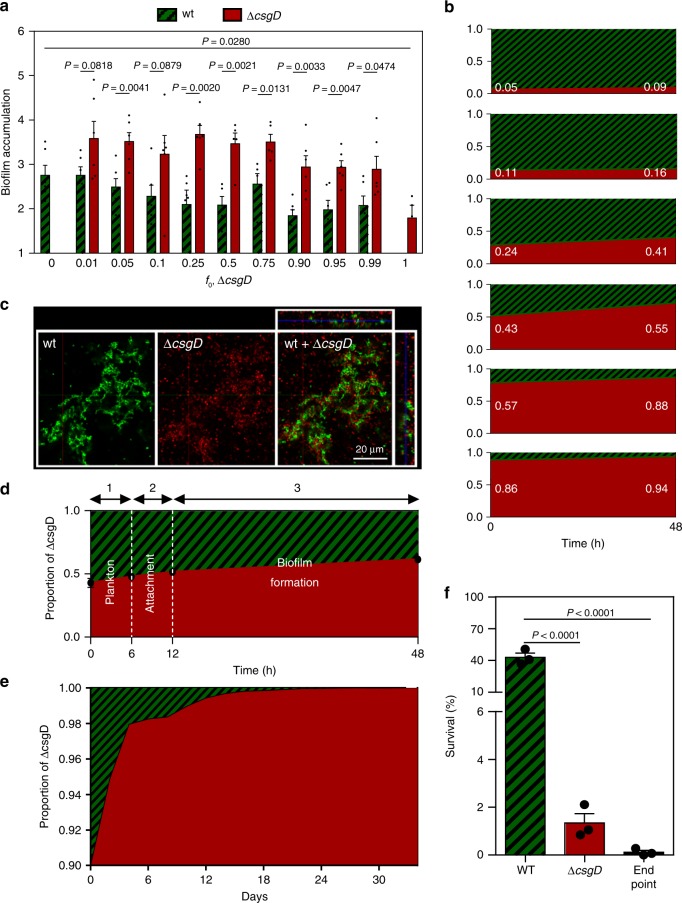
Salmonella biofilm EPS is exploitable. Wild-type strain ATCC14028 (EPS producer) is indicated in (shaded) green; the isogenic Δ*csgD* mutant (EPS non-producer) is indicated in red. **a** Normalised biofilm accumulation of each strain during short-term competition, calculated as $\log _2\frac{{{\mathit{N}}_{{\mathrm{t}} = 48{\mathrm{h}}}}}{{{\mathit{N}}_{{\mathrm{t}} = 0{\mathrm{h}}}}}$; *f*_0, Δ*csgD*_ *=* initial inoculation fraction of Δ*csgD*. **b** Proportion of strains during short-term competition. **c** Confocal image of association between EPS producer and non-producer in the biofilm after 48 h (*f*_0, Δ*csgD*_ *=* 0.9); left: split images, right: combined image. **d** Proportion of strains at different stages of growth during competition in petridishes, 1: plankton before attachment; 2: biofilm during attachment; 3: biofilm after attachment. To exclude interference from attaching cells when studying competition during growth in the biofilm, the planktonic phase above the biofilm was replaced by sterile nutrients at the 12 h time point. **e** Proportion of strains during long-term competition. **f** Survival of endpoint populations of long-term competition after treatment with H_2_O_2_ (0.25%) in comparison with wild-type and Δ*csgD* mutant. For competition experiments **a**, **b**, **d**, **e** and microscopy **c**, wild type and Δ*csgD* mutant were fluorescently labelled in green and red, respectively. A similar outcome **a**, **b**, and **c** was obtained when colors were reversed and competition was neutral when strains that only differ in the fluorescent protein marker were competed against each other (Supplementary Fig. 2). Bars represent mean, dots represent measurements for biological replicates and error bars show s.e.m. (*n* = 3 biologically independent samples, except in Fig. 2a, where *n* = 6). *P* values derived from two-tailed student’s *t* test **a** using Welch’s correction if s.d. are significantly (*P* *<* 0.05) different and one-way ANOVA **f**, with Bonferroni multiple comparisons correction. Source data are provided as a Source Data file.

EPS, therefore, appears to be shared between *Salmonella* cells consistent with a public good. For EPS inhibitors to select against resistance, however, EPS non-producers (equivalent to a strain susceptible to an EPS inhibitor) must be able to benefit so much from shared EPS that they can outcompete producers (equivalent to a resistant strain). We competed wild-type *S*. *Typhimurium* (EPS producer) and the Δ*csgD* mutant (EPS non-producer) across a wide range of initial frequencies (1–99% of mutant) in the biofilm assay (described above) where cells attach to the bottom of petridishes filled with liquid medium (after 6–12 h; Supplementary Fig. 1) and form biofilms for 48 h. As expected, biofilm formation decreases with increasing proportion of the mutant (Supplementary Fig. 3). Moreover, the non-producers benefit greatly from the presence of producers (Fig. 2a). Despite making a poor biofilm in monoculture, the Δ*csgD* mutant outcompetes the wild type across all initial frequencies (Fig. 2a, b). A time course suggests that the Δ*csgD* mutant gains in frequency throughout the 48 h, with the largest proportion of change (53.7%) occurring during growth in the biofilm after attachment (12–48 h) (Fig. 2d). Consistent with the ability of the mutant to exploit the EPS of wild-type cells, the Δ*csgD* mutant specifically populates the same towers formed by wild-type cells in the mature biofilm (Fig. 2c).

The mutant, therefore, outcompetes the wild type at all initial frequencies. However, the most relevant condition for our proof of principle is a biofilm where wild type is rare (simulating a mutant resistant to an EPS inhibitor). We, therefore, focused on a competition where wild type is in the minority (10%) and followed the competition for a month of 48-hour cycles of biofilm formation. Consistent with short-term competition, the wild type declines throughout the experiment falling to below detection after 17 days (< 1 cell per ~ 15,000 Δ*csgD* mutant cells) (Fig. 2e). Importantly, the resulting biofilm lacks structure (Supplementary Fig. 4) and tolerance to antimicrobials (Fig. 2f). In sum, wild-type cells are outcompeted during biofilm formation by non-producers, suggesting again that our strategy to create an antimicrobial where there is natural selection against resistance is tenable.

In related strains and species, CsgD has been shown to regulate a number of additional loci, next to those involved in production of the EPS components cellulose and curli^56–58^. To validate that the relative fitness advantage of the Δ*csgD* mutant in biofilms is specifically related to reduced production of cellulose and curli fimbriae, we compared the Δ*csgD* mutant with a Δ*bcsA*Δ*csgA* double mutant. As expected, the double mutant also has a saw (smooth and white) morphotype (Fig. 1f). Like Δ*csgD*, it also shows reduced biomass in the 48 h biofilm assay (Supplementary Fig. 5a) but increase in growth rate in well-mixed liquid culture (Supplementary Fig. 5b). Furthermore, the double mutant outcompetes the wild type to a similar extent as the Δ*csgD* mutant in the biofilm assay across a wide range of initial frequencies (10–90% of mutant) (Supplementary Fig. 5c). Exploitation of curli fimbriae appears to contribute most strongly to the relative fitness advantage of EPS non-producers given the finding that a Δ*csgA* single mutant outcompetes the wild type to a higher extent than a Δ*bcsA* single mutant (Supplementary Fig. 5d). Consistently, the double mutant is able to outcompete the Δ*bcsA* mutant exploiting its curli production, but not the Δ*csgA* single mutant (Supplementary Fig. 5d). The exploitation of curli fimbriae is unlikely to arise from a direct sharing of CsgA curlin subunits and polymerisation into fimbriae on the surface of the Δ*csgA* mutant, because earlier studies have shown that the wild-type strain (unlike a Δ*csgB* mutant) is not a good donor of CsgA^59,60^. A more likely explanation is that curli non-producers attach to producers and increase their ability to attach, and remain attached, within a biofilm. Consistently, earlier work has shown that curli fimbriae are indispensable for both surface adhesion and cell aggregation^50,61^. Cellulose on the other hand was shown to be less crucial for surface attachment and aggregation, although it strengthens the intercellular interactions^46,50^.

These data are broadly consistent with previous work in *P. fluorescens*^62^ and *Bacillus subtilis*^63^ showing that EPS is a cooperative trait that can be exploited by non-producers. The prospect of evolutionarily robust EPS inhibitors is therefore not limited to *Salmonella*. A potential limitation is that the shareablity—and thus exploitability—of EPS and other public goods has been shown to depend on the population spatial structure and local cell density, with strong lineage segregation and low density specifically confining public goods to producing cells^24,32,39,63–65^. However, it is also important to consider the conditions under which a resistant mutant cell might arise in a biofilm under treatment by a biofilm inhibitor. The focal cell, which would make EPS in spite of the inhibitor, is likely to be surrounded by the non-producer cells it derived from, and therefore subject to competition no matter what the spatiogenetic structure in other parts of the biofilm. However, some EPS types -such as *P. aeruginosa* PSL polysaccharide- appear to be inherently nonexploitable because most fitness benefits accrue to EPS-producing cells^66^. In addition, studies in *P. fluorescence*^67^, *P. aeruginosa*^68^ and *Vibrio cholerae*^69,70^ have shown that certain EPS components specifically evolve to compete with non-producers, by providing superior positioning within the biofilm^25,39^. It is clear, therefore, that not all forms of EPS will be suitable targets for our proposed strategy. However, for EPS in *Salmonella* biofilms, the competition experiments suggest it is indeed a suitable target.

### Resistance does not evolve to an EPS inhibitor

We next sought to put our anti-biofilm strategy where resistance is counter selected into action. 2-cyclopentenyl-5-(4-chlorophenyl)-2-aminoimidazole, a specific member of the class of 5-aryl-2-aminoimidazoles was chosen as EPS inhibitor^34,35^ (see below). We have previously reported that 5-aryl-2-aminoimidazoles prevent EPS production of *S*. *Typhimurium* by reducing the transcription of *csgD* and its regulon^36^. These compounds can be coated to surfaces—both covalently^14^ or in a slow-release matrix^71^ to prevent *Salmonella* biofilm formation in the food industry. Such inhibitors also have clinical potential. For example, an inhibitor might be given prophylactically to inhibit *Salmonella* colonization in the gut when the risk of exposure to *Salmonella* is high, as is currently done with probiotics^72^ and vaccines^73^. The potential for clinical application is further supported by our recent reports showing that 2-aminoimidazoles can inhibit biofilm formation in vivo in a subcutaneous model in rats^14^, have low cytotoxicity against different mammalian cell types (tumor cell lines and bone cells^35,74^) and do not affect the survival of *Caenorhabditis elegans*, a small nematode that is widely used for toxicity testing and is considered to have high predictive value for toxicity in mammals^75^. However, while clinical potential clearly exists, the assays we report here are most reflective of industrial treatments.

**Fig. 3:**
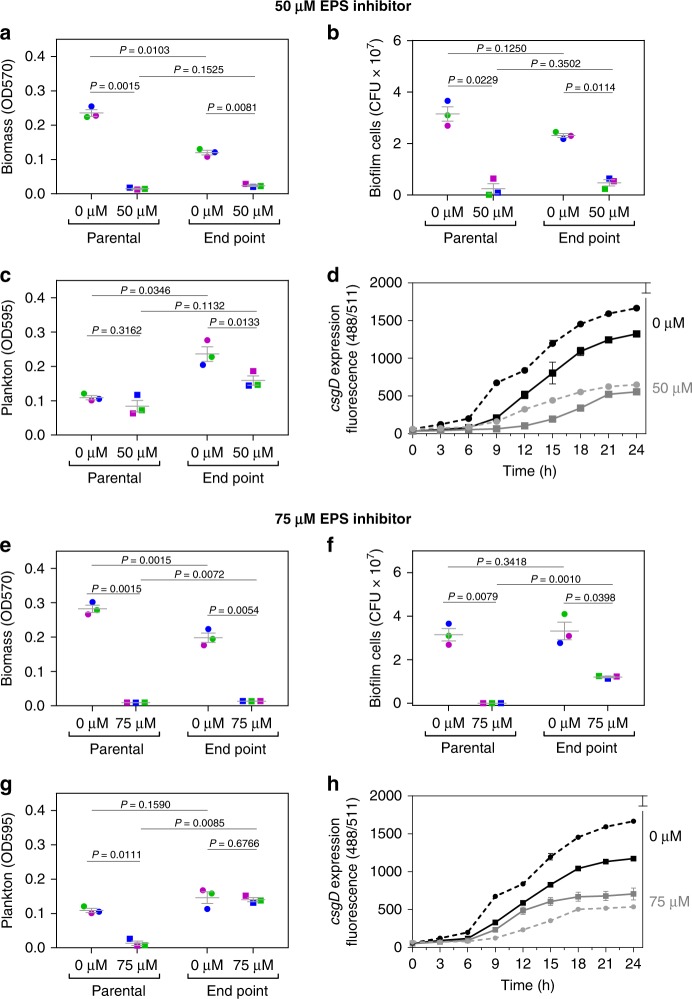
Resistance to EPS inhibition does not evolve over 40 days. Effect of 50 µm EPS inhibitor on **a** biomass, **b** number of biofilm cells, and **c** plankton in well-shaken tubes of parental wild-type strain ATCC14028 and evolved end point populations. **d** *csgD* expression of parental (dashed line) and end point (full line) biofilm strains in function of time in absence and presence of 50 µm EPS inhibitor. || Effect of 75 µm EPS inhibitor on **e** biomass, **f** number of biofilm cells, and **g** plankton in well-shaken tubes of parental wild type strain ATCC14028 and evolved end point populations. **h** *csgD* expression of parental (dashed line) and end point (full line) biofilm strains in function of time in absence and presence of 75 µm inhibitor. Lines represent mean, dots represent measurements for three parallel evolved populations and error bars show s.e.m (*n* = 3 parallel evolution experiments, started from three biologically independent samples). Exceptions are Figures **d** and **h**, which show results for one evolution experiment (one clone isolated from the parental and end point population; tested in three technical repeats), representative for the three parallel evolution experiments. *P* values derived from two-tailed paired student’s *t* test. Source data are provided as a Source Data file.

To further characterize the activity of the selected EPS inhibitor, we studied the effects on *csgD* transcription^26^, biofilm formation and liquid culture growth (to check there is no growth inhibition). At a concentration of 50 µm, *csgD* expression (Fig. 3d) and biofilm formation (Fig. 3a, b) are strongly inhibited, whereas planktonic growth (Fig. 3c) is unaffected. Moreover, residual biofilm formation of the Δ*csgD* mutant is insensitive to the inhibitor, further confirming its activity against curli fimbriae and cellulose (Supplementary Fig. 6).

To test for the evolution of resistance to the EPS inhibitor, we performed a serial passage evolution experiment of wild type *S*. *Typhimurium* in the presence of 50 µm of EPS inhibitor. Biofilms were grown on the bottom of petridishes filled with nutrient-poor liquid and scraped off every 48 h to re-inoculate a clean petridish. We only passaged attached biofilm cells in order to select for biofilm-associated traits as strongly as possible. Evidence that this assay does indeed select for biofilm formation is supported by control experiments performed in the absence of the inhibitor, where we observe an evolutionary response of increased biofilm formation via increased attachment (Supplementary Fig. 7). This experiment was intended to simulate common examples of biofilm contamination in industry. For example, conveyer belts in food industry have biofilms continuously removed with a fixed scraper, but the inability to sufficiently clean this scraper commonly results in re-inoculation of the conveyer belt after cleaning^76^. Over 20 passages in our assay, we observed no change in EPS inhibition (Fig. 3a and Supplementary Fig. 8). This was also reflected at the transcriptional level: *csgD* expression in strains isolated from the last day of the evolution experiment is just as suppressed as in the parental wild type strain (Fig. 3d). Also no increase in biofilm cell count was observed (Fig. 3b), indicating that no alternative strategies for surface attachment have evolved. The only phenotypic change observed during our selection experiment is in untreated cells; these cells evolved to decrease investment into biofilm formation. The only effect of selection in the presence of the inhibitor, therefore, was a decrease in the targeted trait. Consistent with this, we also see *csgD* expression go down (Fig. 3d) and an increase in yield in shaking cultures (Fig. 3c). In sum, no resistance development against the EPS inhibitor occurred and, after 40 days of evolution, the inhibitor-treated biofilm remained highly susceptible to hydrogen peroxide treatment (Supplementary Fig. 9). By contrast, resistance to conventional antimicrobials evolved in a few days in the same experimental setup (Supplementary Fig. 10).

We next explored the effects of a higher concentration of the biofilm inhibitor. At 75 µm, the 2-aminoimidazole inhibits growth in planktonic culture (Fig. 3g) and, therefore, behaves more like a conventional antimicrobial. The absence of biofilm cells on the surface (Fig. 3f) and the low biomass (Fig. 3e) and *csgD* expression levels (Fig. 3h) in the presence of 75 µm inhibitor are thus a result of both EPS inhibition and planktonic growth inhibition. Passaging biofilms under 75 µm 2-aminoimidazole resulted in the evolution of rapid resistance to the growth-inhibitory effects (Fig. 3f and g). What was striking, however, is that we still saw no loss in EPS inhibition over the 40 days. Gene reporter fusion experiments showed that the 2-aminoimidazole still strongly reduces biomass (Fig. 3e) and *csgD* expression (Fig. 3h) in strains isolated from the last day of the evolution experiment. Indeed, the biomass and *csgD* expression levels are the same as those after treatment with 50 µm 2-aminoimidazole (Fig. 3a, d). Consistently, the number of cells on the surface is also still inhibited by 76% (Fig. 3f), equal to the reduction in cell number after treatment with a 50 µm of 2-aminoimidazole (Fig. 3b) or when *csgD* is genetically turned off (Fig. 1b). Even with a single compound, therefore, we can show a clear distinction between conventional resistance evolution via growth inhibition, and our public good strategy where no resistance evolution occurs. This also indicates that an exact dose is not needed to limit resistance evolution because, with a too-high dose, only the off-target effects of excess drug are lost, while anti-biofilm susceptibility is preserved.

### The EPS inhibitor selects against resistant strains

We see no change in sensitivity to our inhibitor, which is consistent with our goal of a strategy where resistance is counter selected. However, the fact that we are unable to select for resistant strains also presents us with a conundrum. Without resistance evolution, we cannot directly demonstrate that resistant strains are outcompeted by susceptible strains. This demonstration is critical to establishing that we have designed an antimicrobial strategy where resistance evolution is counter selected.

**Fig. 4:**
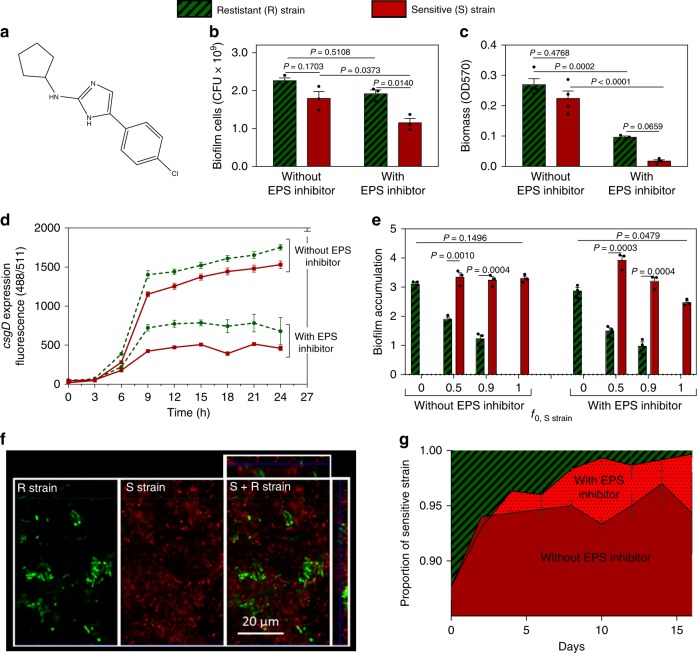
A resistant Salmonella strain (R) is outcompeted by a sensitive strain (S) under inhibitor (50 µm) treatment. Strain resistant to EPS inhibitor (SGSC3068) is indicated in (shaded) green; Strain sensitive to EPS inhibitor (SGSC2227) is indicated in red. **a** Chemical structure of EPS inhibitor 2-cyclopentenyl-5-(4-chlorophenyl)-2-aminoimidazole. **b** Number of cells in monoculture biofilms. **c** Amount of biomass in monoculture biofilms. **d** *csgD* expression of monoculture biofilms of R strain (dashed green line) and S strain (full red line) in function of time. **e** Normalised biofilm accumulation of each strain during short-term competition (*f*_0, S strain_ *=* initial inoculation fraction of the S strain). **f** Confocal image of association between EPS producer and non-producer in the biofilm (*f*_0, S strain_ *=* 0.9); left: split images, right combined image. **g** Proportion of strains during 16 days of competition. Dark and bright red indicate proportion of sensitive strain in biofilms grown in the absence and presence of EPS inhibitor, respectively. For competition experiments **e** and **g** and microscopy **f**, resistant and sensitive strain were fluorescently labelled in green and red, respectively. A similar outcome **e** and **f** was obtained when colors were reversed and competition was neutral when strains that only differ in the fluorescent protein marker were competed against each other (Supplementary Figs. 14, 15a, **c**. Bars and lines represent mean, dots represent measurements for biological replicates and error bars show s.e.m. (*n* = 3 biologically independent samples, except in Fig. 4c, where *n* = 4). *P* values derived from two-tailed student’s *t* test **e** using Welch’s correction if s.d. are significantly (*P* < 0.05) different and two-way ANOVA **b**, **c**, with Bonferroni multiple comparisons correction. Source data are provided as a Source Data file.

We, therefore, pursued non-evolutionary strategies to identify a strain resistant to our EPS inhibitor. First, we screened > 3500 single knockout mutants^77^ of *S*. *Typhimurium*. However, none showed clear resistance to 50 µm of inhibitor. We therefore moved to study natural variation and screened the *Salmonella* reference (SAR) collection of natural isolates^78^. Here, from 151 natural *Salmonella* isolates, we successfully identified two strains that differ greatly in sensitivity to the EPS inhibitor (Fig. 4c). When untreated, EPS production of both strains is similar, although slightly higher for the R strain (Fig. 4b, c). Both strains show a rdar morphotype on CR-agar plates, which indicates that they are producing cellulose and curli fimbriae as EPS components (as was reported before for the R strain^79^) (Supplementary Fig. 11) and targeted sequencing confirms that both encode CsgD. Importantly, their difference in sensitivity to the EPS-inhibitor is reflected in all of biofilm formation, *csgD* transcription (Fig. 4d), and biofilm cell number (Fig. 4b). Upon treatment, the sensitive strain (S) shows an inhibition of biomass, endpoint EPS expression and cell number of resp. 92, 64, and 35%, whereas the resistant strain (R) strain is affected to a lower proportional extent and only shows inhibition levels of resp. 62, 50, and 15% (Supplementary Fig. 12). Residual biofilm formation of a Δ*csgD* mutant of the S strain is insensitive to the inhibitor, further confirming that the inhibitor also targets CsgD in this strain (Supplementary Fig. 13). Moreover, confocal imaging showed less biofilm structure in a treated biofilm of the sensitive strain (S) in comparison with the resistant strain (R) (Supplementary Fig. 14). These two natural strains, therefore, allow us to study competition between sensitive and resistant strains and ask whether applying the inhibitor does indeed select for resistance. It should nevertheless be kept in mind that both strains are non-isogenic and therefore the menu of interaction mechanisms -beyond EPS- is undefined.

We competed the S and R strain during biofilm formation in the petridish assay with and without the 50 µm of EPS inhibitor. Without inhibitor, the S strain has higher fitness than the R strain in mixed culture, across inoculation ratios (Fig. 4e). This is expected because the S strain makes slightly less EPS, even in the absence of inhibitor (Fig. 4c, d). Importantly, treating mixed cultures with the inhibitor does not favour the resistant strain. In fact, the R strain does even worse than in the absence of inhibitor treatment (Fig. 4e), consistent with the more pronounced difference in EPS production (Fig. 4c, d). Moreover, during a long-term competition of seven 48-hour cycles of biofilm formation, the R strain decreases in time in absence of the inhibitor. Again, this effect is only amplified by addition of the EPS inhibitor (Fig. 4g). We also see the expected co-aggregation between the two strains where the S strain is enriched in the biofilm towers formed by the R strain (Fig. 4f and Supplementary Fig. 15). Also in plankton the S strain outcompetes the R strain, as is expected because EPS production is costly here but not beneficial (Supplementary Fig. 15). These data show that, although resistance mechanisms against the EPS inhibitor do exist, a resistant strain is counter selected in the presence of the inhibitor.

The evolution of antimicrobial resistance is threatening a return to the pre-antibiotic era. We urgently need new strategies to inhibit bacteria, particularly strategies where resistance evolution is itself inhibited. We hypothesized that biofilms, where cell–cell interactions are particularly common, are the ideal place to deploy social evolution approaches to limit resistance evolution. In support of this, we have shown that interference with public good cooperation in biofilms is an effective way to treat bacteria, whereas selecting against resistance. Beyond biofilms, public good cooperation remains common in microbes, and important for their biology and societal impacts^12,16,17,34^. This suggests that public good inhibition may be a widely applicable solution to the rising threat of antimicrobial resistance.

## Materials and methods

### Bacterial strains, plasmids, and growth conditions

Experiments were performed using wild-type *Salmonella enterica*, subsp. *enterica* serovar *Typhimurium* ATCC14028^80^, the isogenic ATCC14028 Δ*csgD*, Δ*csgA*, Δ*bcsA*, and Δ*bcsA*Δ*csgA* deletion mutants, and two *Salmonella* strains from the SAR collection (SGSC2227 and SGSC3068)^78^. SGSC2227 (referred to as S) is *Salmonella enterica* subsp. *enterica* serovar Paratyphi B (var. Java) str. CFSAN000529 and was isolated from sewage in Scotland in 1983, whereas the SGS3068 (referred to as R) is *Salmonella enterica* subsp. *diarizonae* str. CFSAN000558 and was isolated from human in Oregon in 1987. In addition, the isogenic SGSC2227 Δ*csgD*, Δ*csgA*, Δ*bcsA,* and Δ*bcsA*Δ*csgA* and the isogenic SGSC3068 Δ*bcsA* where constructed through P22 phage transduction^81^ with the ATCC14028 deletion mutants as donor strains. The deletion mutants in ATCC14028 were constructed through homologous recombination based on the method of Datsenko and Wanner^82,83^. Primers used for constructing the deletion mutants are shown in Supplementary Table 1. To differentiate between the strains during competition experiments, a green fluorescent plasmid (pFPV25.1; *gfp*mut3, Ap^R^) and a red fluorescent plasmid (pFPV25.1; *dsred.T4*, Ap^R^) was used^84^. For in vitro microscopy we made use of the pMax green and red fluorescent protein constructs (Lonza). Cultures were grown overnight (ON) at 37 ˚C in Lysogeny broth (LB) in test tubes with aeration at 200 rpm or on LB plates containing 1.5% agar (w v^−1^). Biofilm assays were performed using a 1/20 dilution of Tryptic Soy Broth (TSB 1/20). Congo red agar (10 g l^−1^ tryptone, 5 g l^−1^ yeast extract, 15 g l^−1^ agar supplemented with 40 µg ml^−1^ congo red and 20 µg ml^−1^ coomassie brilliant blue) was used to study the rdar morphotype. If fluorescently labelled plasmids pFPV25.1 or pMax were present, 100 µg ml^−1^ Ampicillin or 50 µg ml^−1^ kanamycin, respectively, was added to overnight cultures and biofilms. The EPS inhibitor used was 2-cyclopentenyl-5-(4-chlorophenyl)-2-aminoimidazole^34–36^ dissolved in DMSO. The carrier solvent (DMSO) did not have an effect on biofilm formation or planktonic growth at the applied concentration (0.05%).

### Biofilm assay in small petridishes

The OD_595_ of the appropriate ON cultures was measured and corrected to an OD_595_ of 3.2. The corrected cultures were diluted by transferring them into 10 ml TSB 1/20 to obtain an initial cell density of ±12 × 10^7^ ml^−1^. This suspension was poured into small petridishes (Ø 60 mm) and incubated under static conditions at 25 °C. During incubation, the cells attached to and formed a biofilm layer on the bottom of the plates. After 48 h of incubation the liquid medium above the biofilms was removed and 1 ml of phosphate-buffered saline (PBS; 1.24 g l^−1^ K_2_HPO_4_, 0.39 g l^−1^ KH_2_PO_4_, 8.8 g l^−1^ NaCl) was added after which the biofilm layers were scraped off with a cell scraper (Greiner). For the phenotypic switch experiment (Supplementary Fig. 1) parallel biofilm plates were set up and the biofilms formed on the bottom and the side of the petridishes were scraped off every 2 h during 24 h. The biofilm layers were passed five times through a syringe (0.5 × 1.6 mm) and vortexed to disrupt cell clumps and obtain single cells. Biofilm and/or planktonic cells were plated out to determine the number of colony forming units (CFU). Three parallel biological repeats (*n*) were performed, each starting from separate ON cultures.

### Competition experiments in biofilms

Competition was studied between wild-type *S*. *Typhimurium* ATCC14028 (labelled in green) and the isogenic ∆*csgD* mutant (labelled in red) and between the natural isolates SGSC2227 (labelled in red) and SGSC3068 (labelled in green). For short-term competition (48 h), the strains were separately grown in ON cultures, after which the OD_595_ was measured and corrected to an OD_595_ of 3.2. The corrected cultures were diluted and co-inoculated in the appropriate ratios in 10 ml 1/20 TSB, supplemented with EPS inhibitor where needed, to obtain a total cell density ± 12 × 10^7^ ml^−1^ and the exact number of inoculated cells was determined by plate counting. Biofilms were grown in petridishes and scraped off after 48 h as described above. To determine the proportion of each strain at the different stages of biofilm formation (at 6 h, 12 h), parallel biofilm plates were set up and planktonic cells were harvested at 6 h, and biofilm cells scraped off at 12 h. CFUs were counted using an Illumatool Tunable Lighting System (Lightools Research, Encinitas, CA) that allowed to distinguish between green and red fluorescent colonies. The normalised biofilm accumulation of each strain was calculated as the binary logarithm of the number of biofilm cells scraped off at *t* = 48 h divided by the number of cells inoculated at *t* = 0 h:

1 $${\mathrm{Normalised}}\,{\mathrm{biofilm}}\,{\mathrm{accumulation}} = {\mathrm{log}}_2\left( {\frac{{{\mathit{N}}_{{\mathrm{t}} = 48{\mathrm{h}}}}}{{{\mathit{N}}_{{\mathrm{t}} = 0{\mathrm{h}}}}}} \right)$$

The relative fitness of the deletion mutants was calculated as the ratio between the normalised biofilm accumulation of the mutant and the normalized biofilm accumulation of the reference strain.

For long-term competition, an inoculation ratio of 10% EPS producer (wild type strain ATCC14028 or SGSC3068) and 90% EPS non-producer (∆*csgD* mutant or SGSC2227) was used. After 48 h of incubation in petridishes as described above, the liquid medium above the biofilms was removed, after which the biofilms were scraped off and divided into three parts. The first part of 500 µL was used to re-inoculate in 10 ml 1/20 TSB to start a new cycle of biofilm competition in petridishes, the second part (20 µL) was used to make serial dilutions to determine the number of biofilm cells by plate counting, and the third part (480 µl) was stored at − 80 °C. This cycle was repeated 17 times (34 days) and 8 times (16 days), respectively, for competition between wild type strain ATCC14028 and ∆*csgD* mutant and between SGSC3068 and SGSC2227. At least three parallel biological repeats (*n*) were performed, each starting from separate ON cultures.

### Sensitivity to ciprofloxacin and hydrogen peroxide (H_2_O_2_)

Monoculture biofilms of wild-type strain ATCC14028 and isogenic Δ*csgD* mutant were grown in petridishes as described above; cells from three parallel evolved endpoint biofilms were directly inoculated (±8 × 10^6^ cells; from − 80 °C samples) in three technical repeats in 200 µl TSB 1/20 and grown in biofilms on the bottom of 96-well plates. After 48 h of incubation, the liquid medium above the biofilms was removed and fresh growth medium (10 ml or 200 µl TSB 1/20), supplemented with 1 µm ciprofloxacin or 0.25% H_2_O_**2**_, was gently poured onto the biofilms. After 1 additional hour of static incubation at 25 °C, the biofilms were scraped off and the number of cells that survived the treatment were determined by plate counting.

### Rdar colony biofilm assay

In all, 3 µl of the appropriate ON cultures (*S*. *Typhimurium* wild-type strain ATCC14028, Δ*csgA* mutant and Δ*bcsA* mutant) were spotted on CR agar plates after which the plates were incubated statically at 25 °C. After 48 h of incubation, pictures of the rdar colonies were taken using a Canon EOS 450D camera. Three parallel biological repeats (*n*) were performed, each starting from separate ON cultures.

### Crystal violet assay for biofilm biomass determination

To determine the amount of biofilm biomass production of the wild type, the isogenic Δ*csgD* and Δ*bcsA*Δ*csgA* mutants and the two natural isolates SGSC2227 (and its isogenic Δ*csgD* mutant) and SGSC3068 we made use of a crystal violet assay. Also, the screening of the single knock-out mutant library in *S*. *Typhimurium*^77^ and the SAR collection of natural *Salmonella* isolates (Salmonella Genetic Stock Centre)^78^ to identify a strain resistant to the EPS inhibitor (50 µm), was done making use of this crystal violet assay. The OD_595_ of the appropriate ON cultures was measured and corrected to an OD_595_ of 3.2. The corrected ON cultures were diluted 1/100 by transferring them into 1 ml TSB 1/20, supplemented with the appropriate concentration of EPS inhibitor where needed. All knock-out mutants and natural isolates of the mutant library and SAR collection, respectively, were grown separately both in the absence and presence of 50 µm of EPS inhibitor. In all, 200 µl of these suspensions was transferred to the wells of the Calgary Biofilm Device. The lid, a platform bearing 96 polysterene pegs (Nunc number 445497) was placed on a microtiter plate (Nunc number 269787), with a peg hanging into each well. After 48 h of incubation at 25 °C biomass formed on the pegs was measured by crystal violet staining and measuring OD_570_ using a synergy MX multimode reader^85^. Planktonic cell density in the wells of the Calgary Biofilm Device were measured at OD_595_. For the wild type, the Δ*csgD* mutant and the two natural isolates, three parallel biological repeats (*n*) were performed, each starting from separate ON cultures. For the screening of the single knockout mutant library and SAR collection of natural *Salmonella* isolates, one biological repeat was performed.

### Well-mixed liquid media conditions

Growth rate of wild-type *S*. *Typhimurium* ATCC14028 and Δc*sgD* mutant and effect of EPS inhibitor on planktonic growth of parental and end point populations of the evolution experiments, as well as on planktonic competition between the two natural isolates SGSC2227 and SGSC3068, were determined in well-mixed liquid media conditions.

For growth rate determination, the OD_595_ of ON cultures of wild type and Δc*sgD* mutant was measured and corrected to an OD_595_ of 3.2. The corrected cultures were then diluted and co-inoculated in test tubes containing 5 ml TSB 1/20, to obtain a total cell density ± 12 × 10^7^ ml^−1^. The tubes were incubated for 24 h at 25 °C, under vigorously shaking at 300 rpm to avoid clump formation. Every 2 h, the sampled cells were passed five times through a syringe (0.5 × 1.6 mm), followed by plate counting. The relative growth rate of both strains was calculated during late-exponential phase (between 4–20 h), during which *csgD* is expressed. Three parallel biological repeats (*n*) were performed, each starting from separate ON cultures.

For the evolution experiment, cells from the parental strain and parallel endpoint biofilms were directly inoculated (± 8 × 10^6^ cells; from − 80 °C samples) in three technical repeats in 5 ml TSB 1/20 test tubes, supplemented with the appropriate concentration of EPS inhibitor where needed. After 48 h of incubation at 25 °C under shaking conditions (200 rpm), the sampled cells were passed five times through a syringe (0.5 × 1.6 mm), followed by measuring of OD_595_.

For short-term competition (24 h) between the natural isolates SGSC2227 (labelled in red) and SGSC3068 (labelled in green), the strains were separately grown in ON cultures, after which the OD_595_ was measured and corrected to an OD_595_ of 3.2. The corrected cultures were diluted and co-inoculated in the appropriate ratios in 5 ml 1/20 TSB, supplemented with EPS inhibitor where needed, to obtain a total cell density ± 12 × 10^7^ ml^−1^ and the exact number of inoculated cells was determined by plate counting. The tubes were incubated for 24 h at 25 °C, under vigorously shaking at 300 rpm to avoid clump formation. Three biological repeats (*n*) were performed starting from separate ON cultures, and each containing three technical repeats. After 24 h, the sampled cells were passed five times through a syringe (0.5 × 1.6 mm), followed by plate counting. CFUs were counted using an Illumatool Tunable Lighting System (Lightools Research, Encinitas, CA) that allowed to distinguish between green and red fluorescent colonies. The normalised planktonic cell accumulation of each strain was calculated as the binary logarithm of the number of cells at *t* = 24 h divided by the number of cells inoculated at *t* = 0 h:

2 $${\mathrm{Normalised}}\,{\mathrm{planktonic}}\,{\mathrm{cell}}\,{\mathrm{accumulation}} = {\mathrm{log}}_2\left( {\frac{{{\mathit{N}}_{{\mathrm{t}} = 24{\mathrm{h}}}}}{{{\mathit{N}}_{{\mathrm{t}} = 0{\mathrm{h}}}}}} \right)$$

### Serial passage evolution experiments

Serial passage evolution experiments were conducted to evaluate resistance development against the 2-aminoimidazole inhibitor (50 µm and 75 µm), spectinomycin (1 mm) and ciprofloxacin (0.06 µm). A control evolution in the absence of treatment was also included. For each treatment type, three parallel evolution experiments were performed, starting from separate colonies of *S*. *Typhimurium* ATCC14028. The colonies were inoculated and grown ON in LB. The OD_595_ of the cultures was measured and corrected to an OD_595_ of 2.5. The corrected cultures were diluted 1/200 by transferring them into 5 ml TSB 1/20 in the presence of inhibitor or classic antibiotic. These suspensions were poured into small petridishes and incubated at 25 °C under static conditions. After 48 h the biofilm layers were scraped off and divided into four parts. The first part of 500 µl was directly re-inoculated into the following cycle of biofilm formation in petridishes, the second part (20 µl) was used to determine the number of biofilm cells by plate counting, the third part (440 µl) was stored at − 80 °C and the last part (20 µl) was used for biomass/sensitivity evaluation. Hereto, the cells were directly inoculated in 1 ml TSB 1/20 and biomass production was determined in the Calgary Biofilm Device by crystal violet staining, both in the presence and absence of the appropriate concentration of EPS inhibitor (at least three technical repeats), as described above. The entire cycle was repeated 20 times (40 days).

To determine the effect of EPS inhibitor on the number of biofilm cells of the parental and end point populations, cells from the parental strain and parallel endpoint biofilms were directly inoculated (±8 × 10^6^ cells ml^−1^; from −80 °C samples) 3 technical repeats in 200 µl TSB 1/20 in 96-well plates supplemented with the appropriate concentration of EPS inhibitor, where needed. The plates were incubated statically at 25 °C and after 48 h, the biofilm layers were scraped off and passed 5 times through a syringe (0.5 × 1.6 mm), followed by plate counting.

### *csgD* gene expression measurements

Expression of *csgD* was measured by using a transcriptional *gfp*mut3-promoter fusion plasmid^36^. A promoterless *gfpmut3* plasmid was used as a negative control, whereas a constitutive *gfp*mut3-promoter (*rpsM* promoter) plasmid was used as a positive control^36^. Single colonies of three parental strain and three evolved endpoint populations were isolated and the *gfp*mut3-promoter fusion plasmids were introduced into the strains by electroporation. The OD_595_ of the appropriate overnight cultures were measured and corrected to an OD_595_ of 3.2. The cells were diluted 1/100 when transferring them into 1 ml TSB 1/20, supplemented with the appropriate concentration of EPS inhibitor where needed. In all, 200 µl of the cell suspensions were transferred in three technical repeats to a black, polystyrene, clear bottomed 96-well plate (Greiner, Bio-one 655096) and the plates were incubated statically at 25 °C for 24 h. Green fluorescence (excitation 488 nm, emission 511 nm) and absorbance at 600 nm were measured every 3 h using a synergy MX multimode reader. Data were processed as described before^36^.

### Microscopy on in vitro biofilms

Biofilms were grown as described earlier. The plankton above the biofilm was gently poured off and the biofilm was washed with 10 ml PBS to visualize with confocal microscopy. Confocal imaging was carried out on a LSM 700 laser scanning microscope (Zeiss) using the × 50 objective and the associated Zen software.

### Bioscreen for growth rate determination

Growth curves of wild-type *S*. *Typhimurium* ATCC14028 and Δ*bcsA*Δ*csgA* mutant were determined using a Bioscreen C system. The OD_595_ of the cultures was measured and corrected to an OD_595_ of 2.5. The corrected cultures were diluted 1/1000 by transferring them into 10 mL TSB 1/20. In all, 32 technical replicates with 200 µL of the cells were grown at 25 °C with continuous shaking for 48 h. The maximum specific growth rate was calculated from the growth curve using the Gompertz equation^86^.

### Statistical analysis of in vitro assays

All data shown here were collected from at least three parallel biological cultures (*n*), except for microscopic pictures and *csgD* expression profiles of parental strains and end point strains. Data were either analysed by unpaired student’s *t* test using Welch’s correction if s.d. are significantly (*P* < 0.05) different, by paired student’s *t* test or by one-way/two-way analysis of variance with Bonferroni multiple comparisons corrections.

### Reporting summary

Further information on research design is available in the Nature Research Reporting Summary linked to this article.

## Supplementary information

Supplementary Information

Peer Review File

Reporting Summary

## Data Availability

The authors declare that the main data supporting the findings of this study are available within the article and its Supplementary Information. In addition, the source data underlying Figs. 1a–d, 2a, b, d–f, 3a–h and 4b–e, g and Supplementary Figs. 1a, b, 2c, 3, 5a–d, 6a, b, 7, 8a, b, 9, 10a, b, 12a–c, 13 and 15c, d are provided as a Source Data file. Further information and requests for resources and reagents should be directed to and will be fulfilled by Hans Steenackers (hans.steenackers@kuleuven.be). Genomic mutants and plasmids used in these studies can be made available upon request following the signing of a material transfer agreement.

## Source Data

Source Data
