# Supplementary material for: Inhibiting bacterial cooperation is an evolutionarily robust anti-biofilm strategy
Source: Nat Commun. 2020 Jan 9;11:107. doi: 10.1038/s41467-019-13660-x (PMC6952394; doi:10.1038/s41467-019-13660-x)
Supplement: Supplementary file 1 — Supplementary Information [file 41467_2019_13660_MOESM1_ESM.pdf]

Inhibiting bacterial cooperation is an evolutionarily robust anti-biofilm strategy

Supplementary Information

Dieltjens et al.

Supplementary Information

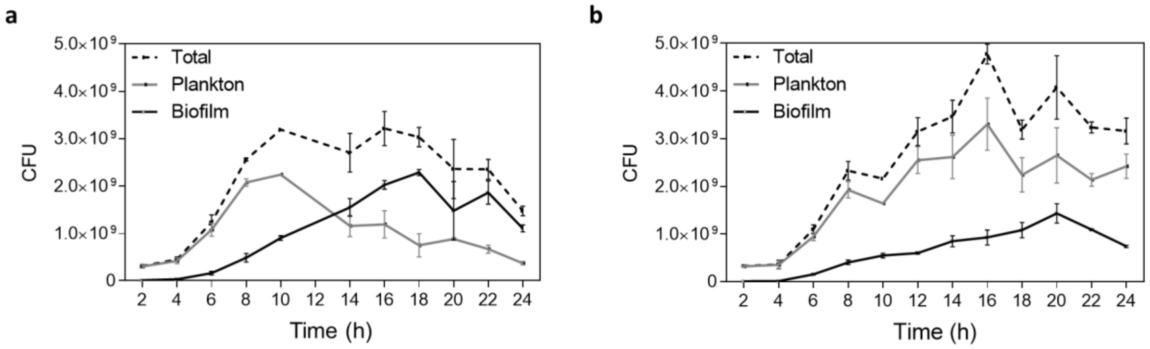

Supplementary Figure 1: Phenotypic switch from planktonic to biofilm state during monoculture growth in petridishes. a) *Salmonella* wild type strain ATCC14028. b) Isogenic Δ*csgD* mutant**.** Lines represent mean and error bars show s.e.m. (n=3, cell culture biological replicates). Source data are provided as a Source Data file.

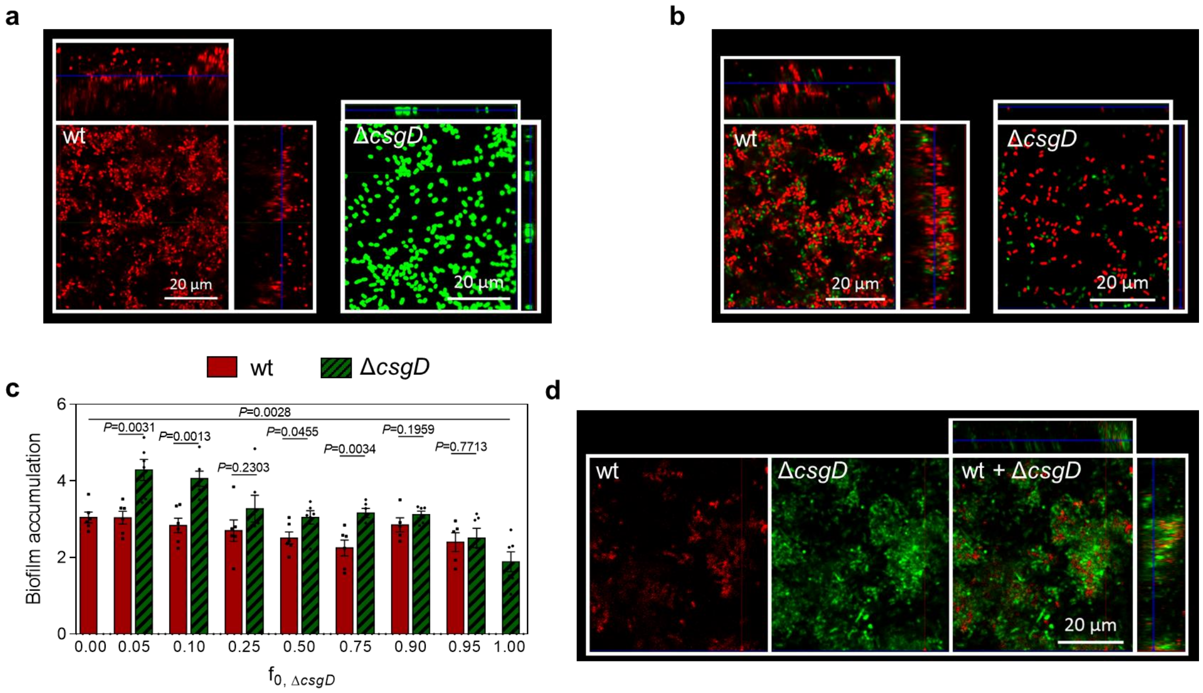

Supplementary Figure 2: Differential fluorescent labelling of wild type strain ATCC14028 and isogenic Δ*csgD* mutant does not affect experimental outcome. a) Confocal images of monoculture biofilms of wild type labelled in red and mutant labelled in green (reversed labelling compared to Figure 1e). b) Confocal images showing identical behaviour of differentially labelled wild type strains (left) and mutant strains (right) when grown in 1:1 competition. c) Normalised biofilm accumulation of wild type strain labelled in red and Δ*csgD* mutant labelled in shaded green during short-term competition (reversed labelling compared to Figure 2A). d) Confocal image of association between wild type labelled in red and Δ*csgD* mutant labelled in green in the biofilm ($f_{0,\ \Delta csgD}$ = 0.9; left split images, right combined image; reversed labelling compared to Figure 2c). Bars represent mean, dots represent measurements for biological replicates and error bars show s.e.m. (n=5 biologically independent samples). P values derived from two-tailed student's t-test using Welch's correction if s.d. are significantly ($P < 0.05$) different. Source data are provided as a Source Data file.

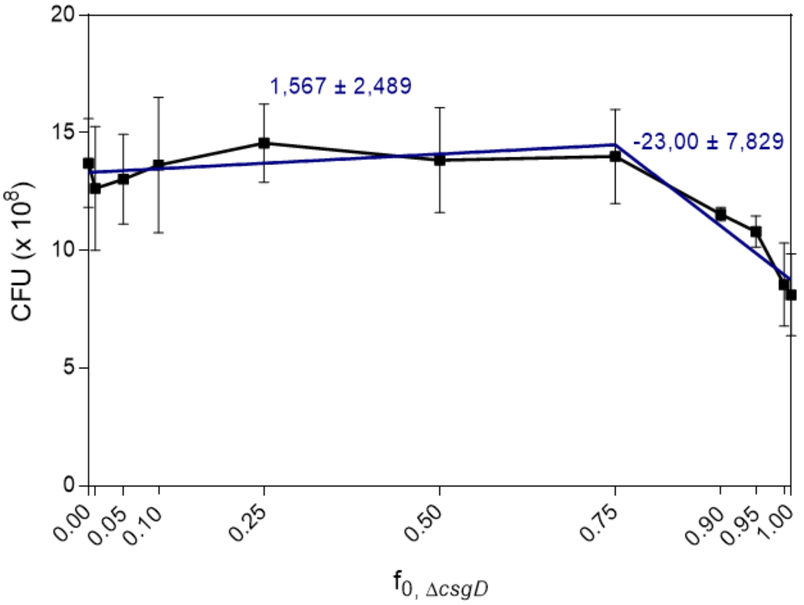

Supplementary Figure 3: Total number of cells during short-term competition between wild type strain ATCC14028 and isogenic Δ*csgD* mutant. Total number of cells consist of wild type and Δ*csgD* mutant cells in the biofilm, $f_{0,\,\Delta csgD}$ = initial inoculation fraction of Δ*csgD* mutant. Dots represent mean of biological replicates and error bars show s.e.m. (n=6 biologically independent samples). A segmental linear regression (blue curve) was performed with constraint $x_0 = 0.75$, yielding a $slope_1$ (from x=0.00 to x=0.75) of 1.576 (s.d. = 2.489, n=6) and a $slope_2$ (from x=0.75 to x=1.00) of -23.00 (s.d. = 7.829, n=6). A one sample t-test indicates that $slope_1$ is not significantly different from zero (P value = 0.1837), whereas $slope_2$ is significantly different from zero (P value = 0.0008). This indicates that at low wild type frequencies the total number of cells in the biofilm decreases with increasing inoculation fraction of Δ*csgD*. Source data are provided as a Source Data file.

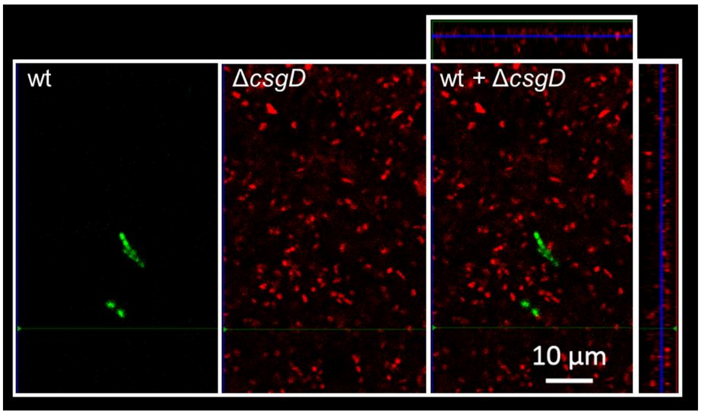

Supplementary Figure 4: Confocal image shows very limited biofilm structure at low EPS producer proportions. *Salmonella* wild type strain ATCC14028 labelled in green, isogenic Δ*csgD* mutant in red ($f_{0,\ \Delta csgD}$ = 0.99; left split images, right combined image).

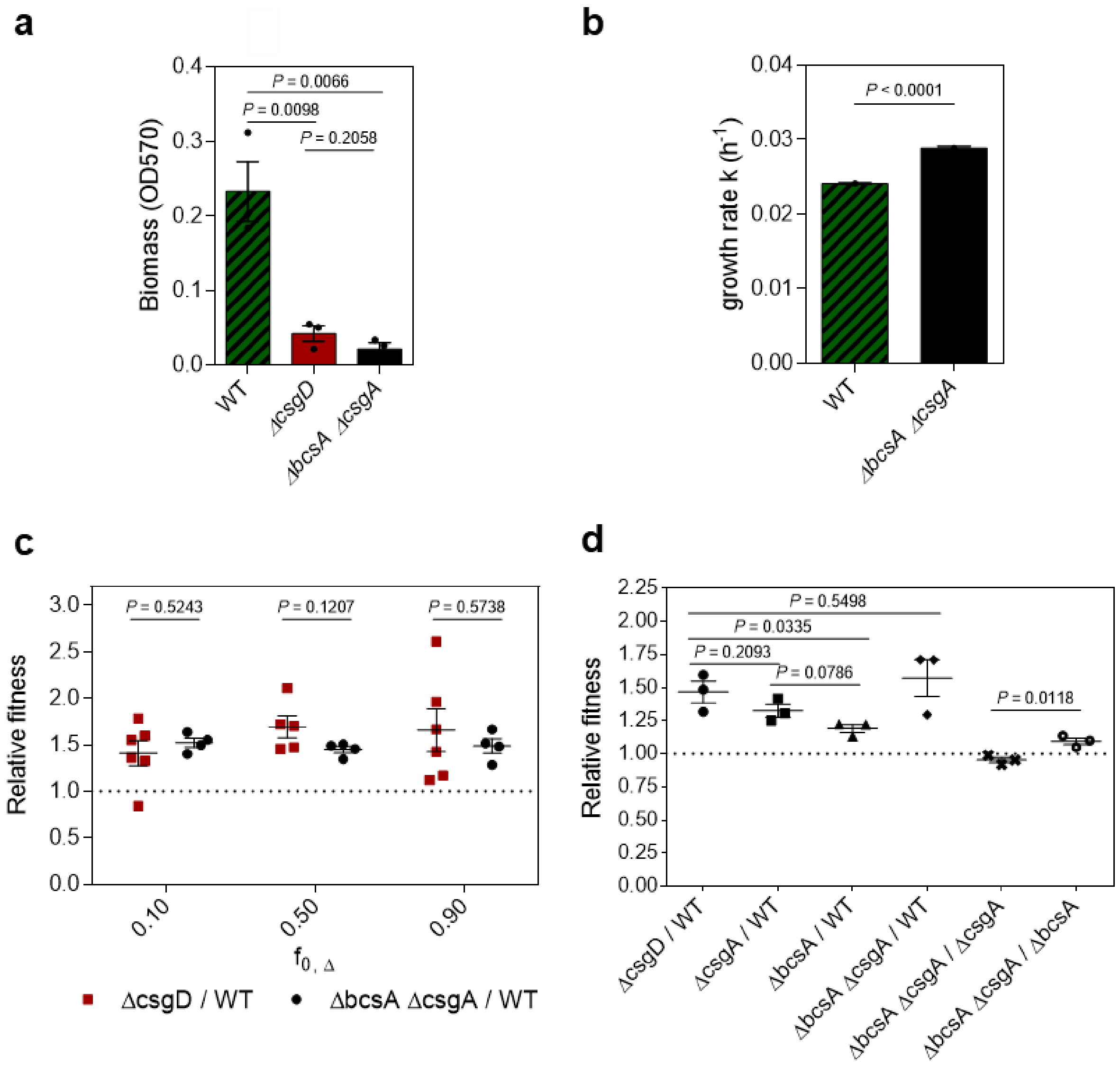

Supplementary Figure 5: The isogenic Δ*bcsA*Δ*csgA* mutant confirms that *Salmonella* biofilm EPS is an exploitable public good. Wild type *S*. Typhimurium strain ATCC14028 (EPS producer) is indicated in shaded green; the isogenic Δ*csgD* mutant (EPS non-producer) is indicated in red; the isogenic Δ*bcsA*Δ*csgA* mutant (EPS non-producer) is indicated in black. a) Amount of biomass in monoculture biofilms. b) Maximum specific growth rate (OD_{600} h^{-1}) during exponential phase in liquid (Bioscreen C system). c) Relative fitness of the mutants during short-term competition with the wild type. The relative fitness is calculated as the ratio between the normalised biofilm accumulation of the mutant and the normalised biofilm accumulation of the wild type, with the normalised biofilm accumulation calculated as $\log_2 \frac{N_{t=48h}}{N_{t=0h}}$; $f_{0,\Delta}$ = initial inoculation fraction of Δ*csgD* or Δ*bcsA*Δ*csgA*. d) Relative fitness of the mutants during short-term competition with the wild type and Δ*bcsA*Δ*csgA* mutant. The relative fitness is calculated as the ratio between the normalised biofilm accumulation of the mutant and the normalised biofilm accumulation of the wild type or the ratio between the normalised biofilm accumulation of the Δ*bcsA*Δ*csgA* mutant and the normalised biofilm accumulation of the wild type, Δ*csgA* and Δ*bcsA* mutant, respectively; the normalised biofilm accumulation is calculated as $\log_2 \frac{N_{t=48h}}{N_{t=0h}}$. Bars and lines represent mean, dots represent measurements for biological replicates and error bars show s.e.m. (n=3 biologically independent samples, except in Figure S5c, where n=4 for Δ*bcsA*Δ*csgA*/WT and n=6 for Δ*csgD*/WT). P values derived from two-tailed student's t-test using Welch's correction if s.d. are significantly ($P < 0.05$). Source data are provided as a Source Data file.

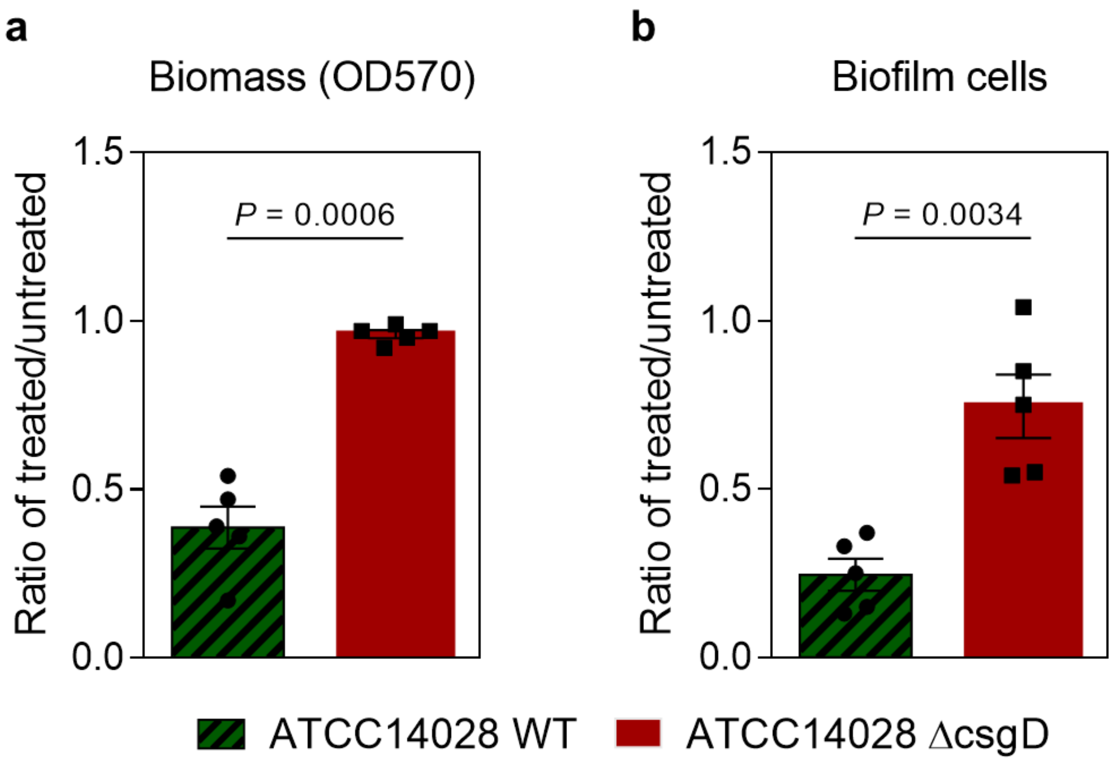

Supplementary Figure 6: The EPS inhibitor acts through inhibition of CsgD in *Salmonella* ATCC14028. Proportional inhibition upon treatment with EPS inhibitor of: a) Biomass and b) Biofilm cells in the *Salmonella* ATCC14028 wild type and isogenic Δ*csgD* mutant strain. Bars represent mean, dots represent measurements for biological replicates and error bars show s.e.m. (n=5 biologically independent samples). P values derived from two-tailed student's t-test using Welch's correction if s.d. are significantly ($P < 0.05$) different. Source data are provided as a Source Data file.

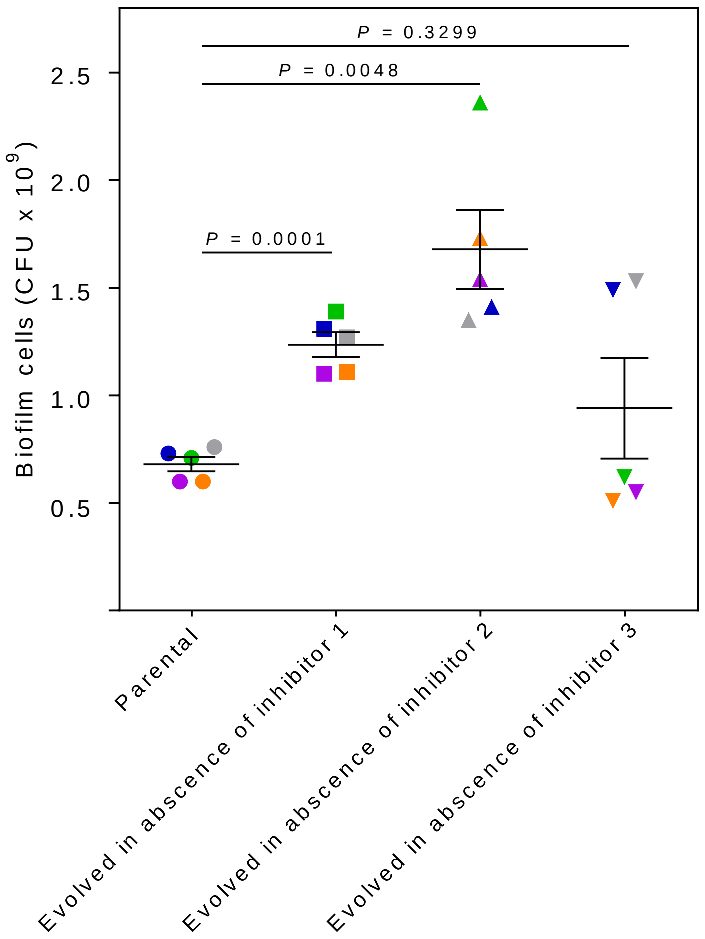

Supplementary Figure 7. Control evolution experiments in the absence of EPS inhibitor show an evolutionary response of increased biofilm formation via increased attachment. Number of cells in biofilms formed by the parental wild type strain ATCC14028 and evolved endpoint strains isolated from 3 parallel populations evolved for 40 days in the absence of EPS inhibitor. Lines represent mean, dots represent measurements for each isolated strain (n=5 biologically independent endpoint populations, except for the parental strain, where n=5 biologically independent samples) and error bars show s.e.m. P values derived from two-tailed paired t-test using Welch's correction if s.d. are significantly ($P < 0.05$) different. Source data are provided as a Source Data file.

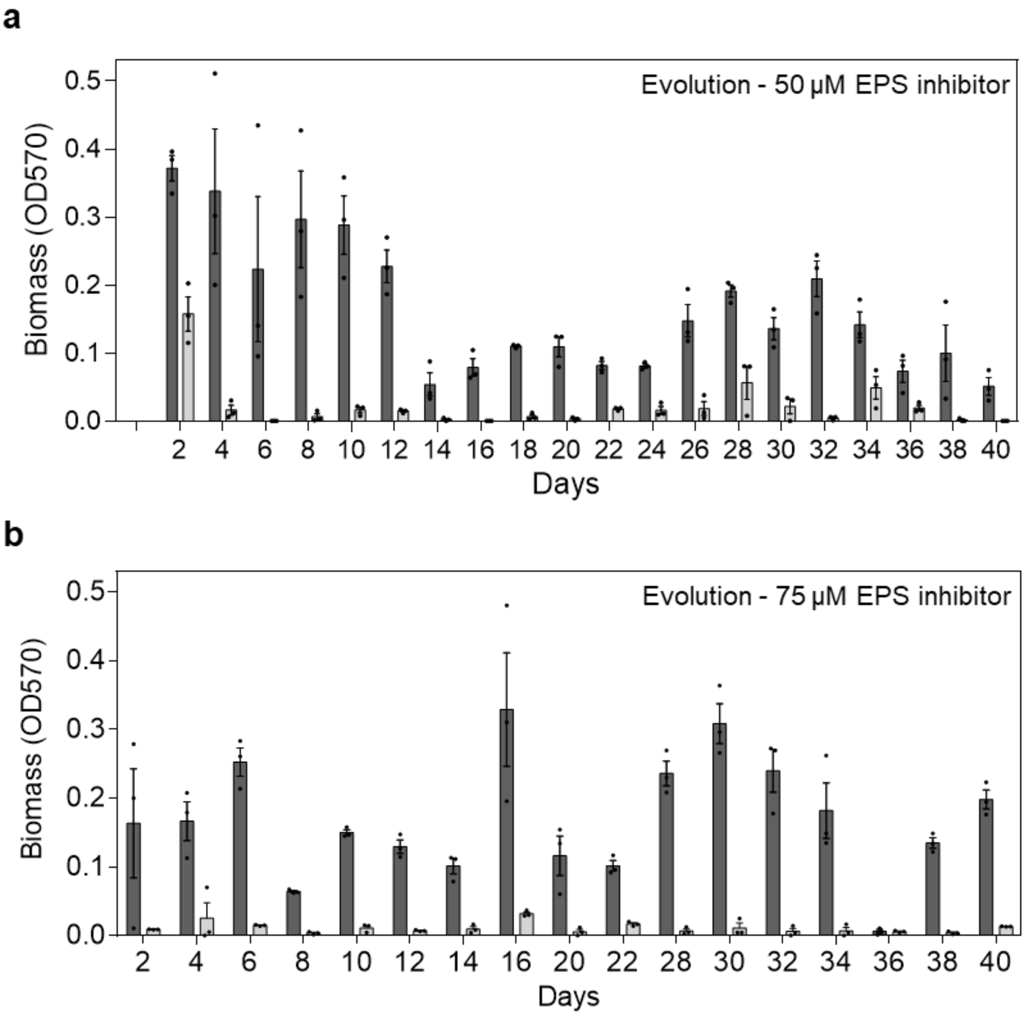

Supplementary Figure 8: Amount of biomass formed by evolving population in function of time, evaluated in absence (dark grey bars) and presence (light grey bars) of EPS inhibitor. a) Evolving population of evolution experiment with 50 µM of EPS inhibitor. b) Evolving population of evolution experiment with 75 µM of inhibitor. Bars represent mean, dots represent measurements for 3 parallel evolved populations and error bars show s.e.m (n=3 parallel evolution experiments, started from 3 biologically independent samples). Source data are provided as a Source Data file.

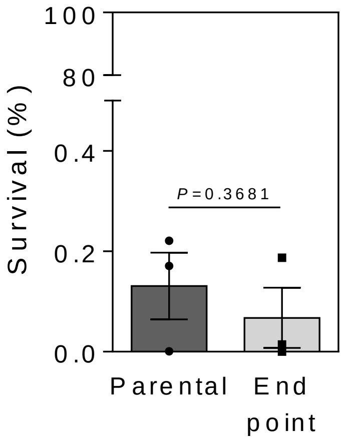

Supplementary Figure 9: Survival after treatment with H_2O_2 (0.25%) of mature biofilms formed by the parental strain and end point populations grown in the presence of 50 µM the EPS inhibitor. Bars represent mean, dots represent measurements for biological replicates and error bars show s.e.m. of 3 parallel evolved populations (n=3 parallel evolution experiments, started from 3 biologically independent samples). P value derived from a two-tailed paired t-test (n=3). No significant difference could be demonstrated ($P > 0.05$). Source data are provided as a Source Data file.

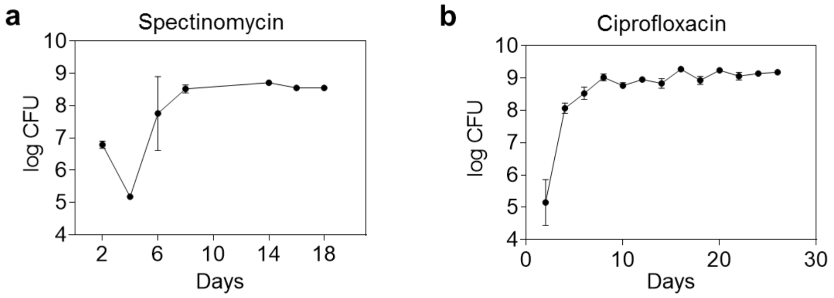

Supplementary Figure 10: Resistance development against antibiotic-like activity readily occurs. a) Number of biofilm cells during evolution in the presence of spectinomycin (1 mM). b) Number of biofilm cells during evolution in the presence of ciprofloxacin (0.06 µM). Bars represent mean and error bars show s.e.m. of 3 parallel evolved populations (n=3 parallel evolution experiments, started from 3 biologically independent samples). Source data are provided as a Source Data file.

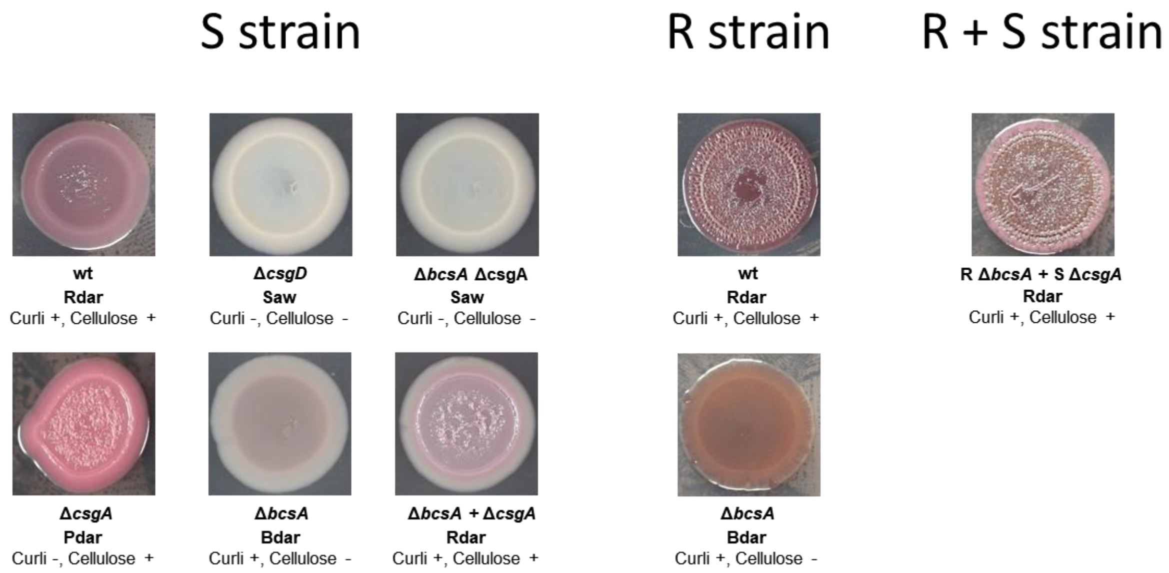

Supplementary Figure 11: Colony morphologies of the wild type and different biofilm mutants of the resistant strain R (SGSC3068) and sensitive strain S (SGSC2227). All mutants show the expected morphotypes on CR-agar plates further validating the role of curli fimbriae and cellulose and CsgD regulation in these strains. Mixing a Δ*bcsA* and Δ*csgA* mutant of the S strain restores its rdar morphotype, confirming that curli and cellulose are shared in this strain. Also mixing Δ*bcsA* mutant of the R strain with the Δ*csgA* mutant of the S strain restores the rdar morphotype indicating the EPS components are also shared between both strains.

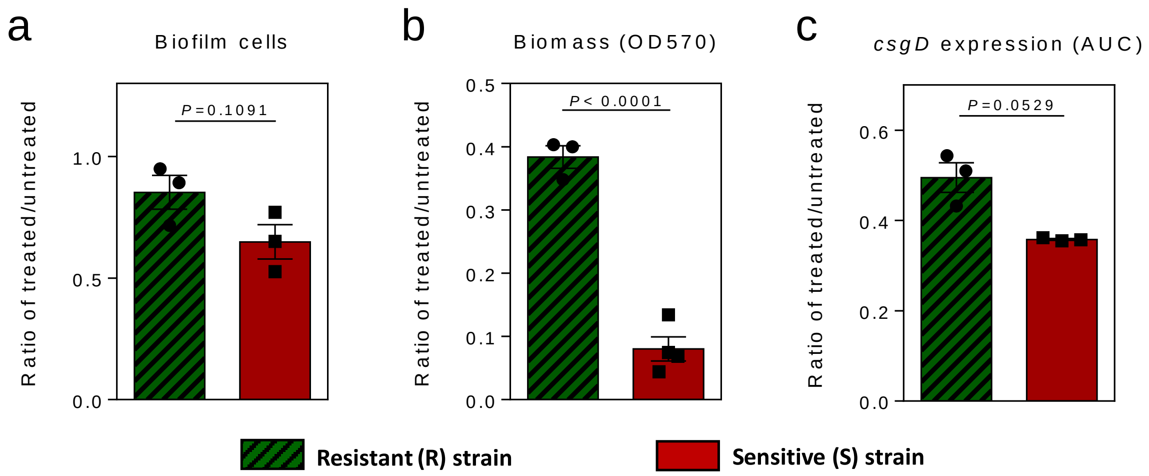

Supplementary Figure 12: Proportional inhibition upon treatment with EPS inhibitor of: a) Biofilm cells, b) Biomass and c) CsgD expression (Area Under Curve) in the resistant (R) and sensitive (S) *Salmonella* strain. Bars represent mean, dots represent measurements for biological replicates and error bars show s.e.m. (n=3 biologically independent samples, except in Figure S12b, where n=4 for the S strain). P values derived from two-tailed student's t-test using Welch's correction if s.d. are significantly ($P < 0.05$) different. Source data are provided as a Source Data file.

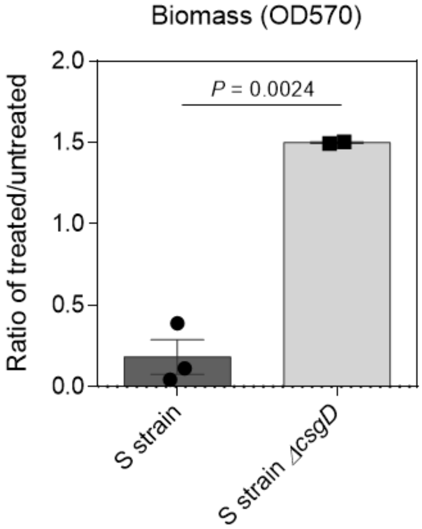

Supplementary Figure 13: The EPS inhibitor acts through inhibition of CsgD in the sensitive strain S (SGSC2227). Proportional inhibition upon treatment with EPS inhibitor of biomass in the sensitive strain S (SGSC2227) wild type and isogenic Δ*csgD* mutant strain. Bars represent mean, dots represent measurements for biological replicates and error bars show s.e.m. (n=3 biologically independent samples for the S strain and n=2 for S strain Δ*csgD*). P value derived from two-tailed student's t-test using Welch's correction if s.d. are significantly ($P < 0.05$) different. Source data are provided as a Source Data file.

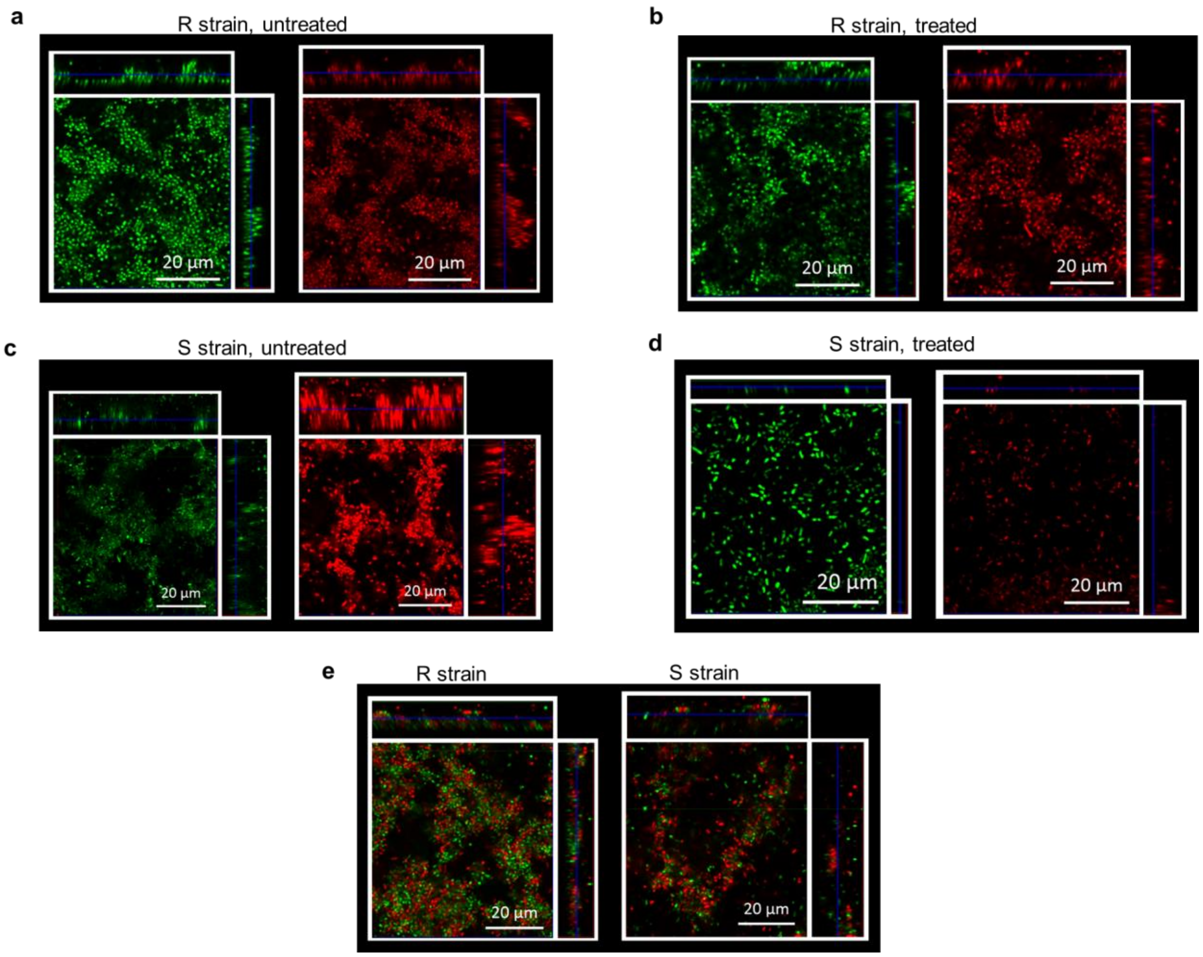

Supplementary Figure 14: Confocal images of differentially labelled monoculture biofilms formed by resistant strain R (SGSC3068) and sensitive strain S (SGSC2227) in absence and presence of 50 µM EPS inhibitor. a) R strain, untreated. b) R strain, treated. c) S strain, untreated. d) S strain, treated. e) Identical behaviour of differentially labelled R strains (left) and S strains (right) when grown in 1:1 competition in absence of inhibitor.

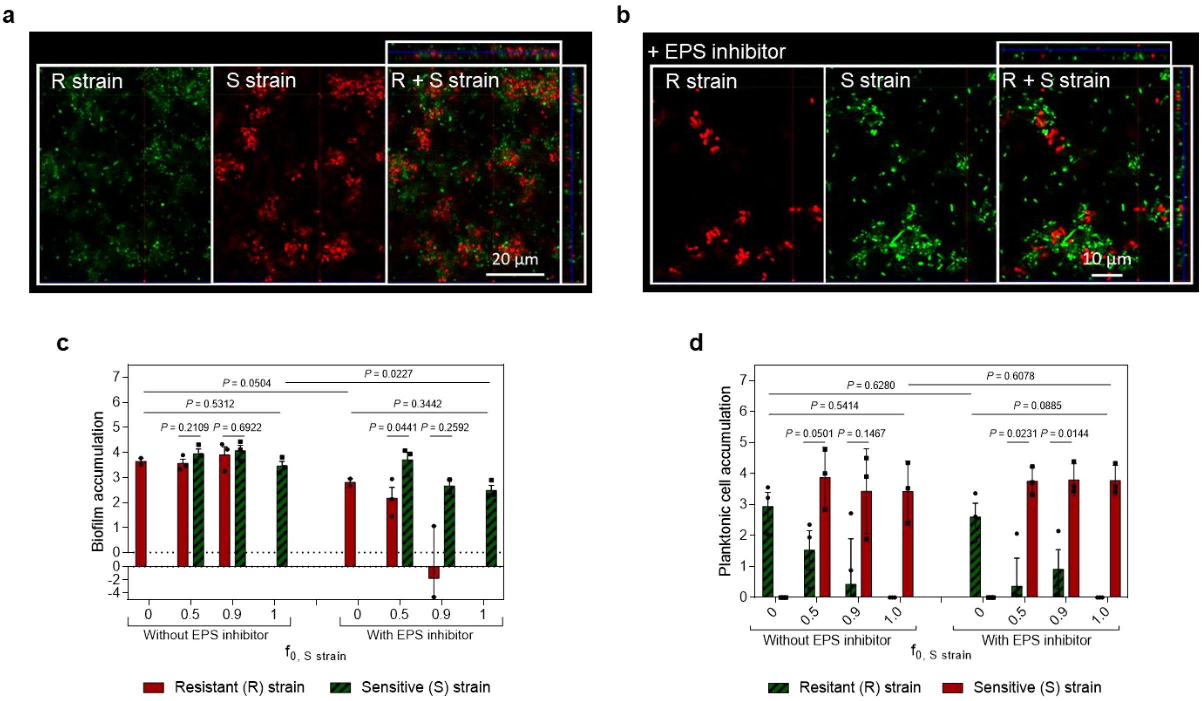

Supplementary Figure 15: Short-term competition of resistant strain R (SGSC3068) and sensitive strain S (SGSC2227). a) Confocal image of association between R strain labelled in green and S strain labelled in red in the biofilm, in the absence of the inhibitor ($f_{0,\, S\ strain}$ = 0.9; left split images, right combined image). b) Confocal image of association between R strain labelled in red and S strain labelled in green in the biofilm in the presence of EPS inhibitor ($f_{0,\, S\ strain}$ = 0.9; split images, right combined image; reversed labelling compared to Figure 4f). c) Normalised biofilm accumulation of R strain labelled in red and S strain labelled in shaded green during short-term competition (reversed labelling compared to Figure 4e). d) Normalised planktonic cell accumulation of R strain labelled in shaded green and S strain labelled in red during short-term competition in plankton in test tubes under shaking conditions. Under these conditions, the two strains behave as expected according to our model for their behaviour under biofilm conditions. Specifically, the sensitive strain which makes less EPS without the inhibitor outcompetes the resistant strain in absence of inhibitor. And this effect becomes more pronounced in the presence of inhibitor which reduces EPS more in sensitive than the resistant strain (Figure 4e). In competition then, the outcome is the same as in biofilms but importantly in monoculture, the sensitive strain reaches a higher cell density than the resistant strain, which differs from biofilm conditions where it is the resistant strain which makes more EPS that does better. These results are as expected because EPS production in plankton is costly but not beneficial, such that the strain that makes the least EPS is expected to reach the highest cell number. Bars represent mean, dots represent measurements for biological replicates and error bars show s.e.m. (n=3 biologically independent samples, except in Figure S15c, where n=2 for $f_{0,\, S\ strain}$ = 0 without and with EPS inhibitor and $f_{0,\, S\ strain}$ = 0.9 with EPS inhibitor). P values derived from two-tailed student's t-test using Welch's correction if s.d. are significantly (P < 0.05) different. Source data are provided as a Source Data file.

Name	Sequence 5' → 3'	Description
pro-6637	ATTCTAGAGCGTCGAAACAGCCGTTAGG	Forward primer for *bcsA* in *S*. Typhimurium ATCC14028
pro-6638	ATGGATCC CAGCGCCATACTACCCGGCG	Reverse primer for *bcsA* in *S*. Typhimurium ATCC14028
S&P-00921	TCAATCCGATGGGGGTTTTAC	Forward primer for *csgA* in *S*. Typhimurium ATCC14028
S&P-00922	TTTTATTAGCGCAGACGCTA	Reverse primer for *csgA* in *S*. Typhimurium ATCC14028
pro-2093	AGTAACTCTGCTGCTACAATCCAGG	Forward primer for *csgD* in *S*. Typhimurium ATCC14028
pro-2094	CCTTTATTTTATGGGGGCAGCTGTCA	Reverse primer for *csgD* in *S*. Typhimurium ATCC14028
S&P-00401	CTATTACCGCCGCACACATCCAGGACAATTTTCT TTTCATCGCATTATCAGTGTAGGCTGGAGCTGCTTC	Forward Datsenko & Wanner primer for *bcsA* in *S*. Typhimurium ATCC14028
S&P-00402	TGGTGCCTGCTGCATGATGCGGGCGACAAAACGT CCGCCGGGAGCCTGCGTCATATGAATATCCTCCTA	Reverse Datsenko & Wanner primer for *bcsA* in *S*. Typhimurium ATCC14028

Supplementary Table 1: Primers used in this study. Primers used for constructing the ATCC14028 Δ*csgD*, Δ*csgA*, Δ*bcsA* and Δ*bcsA*Δ*csgA* deletion mutants, as well as for validating the SGSC2227 Δ*csgD*, Δ*csgA*, Δ*bcsA*, Δ*bcsA*Δ*csgA* and SGSC3068 Δ*bcsA* deletion mutants.
