# Supplementary material for: Inhibiting bacterial cooperation is an evolutionarily robust anti-biofilm strategy
Source: Nat Commun. 2020 Jan 9;11:107. doi: 10.1038/s41467-019-13660-x (PMC6952394; doi:10.1038/s41467-019-13660-x)
Supplement: Supplementary file 2 — Peer Review File [file 41467_2019_13660_MOESM2_ESM.pdf]

Reviewers' Comments:

Reviewer #1:
Remarks to the Author:
The paper by Dieltjens et al. examines whether treatments inhibiting biofilm formation in bacterial pathogens is an evolutionarily robust strategy, where resistant mutants cannot spread in the population. Biofilm formation is based on the secretion of matrix components, which are shared as 'public good' at the level of the group. The authors argue that treatments inhibiting the production of such biofilm matrix components could be evolutionarily robust, because resistant phenotypes would involve the resumption of matrix production, which is costly to the individual cell, but generates benefits to everyone in the group, including the susceptible cells.

To test their hypotheses, the authors conducted a series of experiments with Salmonella typhimurium and the biofilm inhibitor 2-cyclopentenyl-5-(4-chlorophenyl)-2-aminoimidazole. They show that:

a) Matrix components are public goods that are costly to produce for the individual cell, but benefit the group as a whole (fig. 1).

b) Matrix non-producers can exploit matrix producers at all mixing ratios, and increase in frequency, thereby reducing biofilm formation and bringing cooperation to collapse (fig. 2).

c) Resistance against the anti-biofilm inhibitor does not evolve in in-vitro batch-culture experiments (fig. 3).

d) A resistant mutant cannot spread under treatment (fig. 4).

This is a nice and concise piece of work. Similar treatments and concepts have been applied to target cooperative siderophore secretion and quorum-sensing based communication in bacteria, but this is, to my knowledge, the first study that applies the social evolution concept to biofilm treatments. While the paper is very well written and the logic of the arguments are clear and easy to follow, I have a number of points I would like to see addressed:

1) The authors put forward strong arguments regarding the evolutionary robustness of treatments targeting social traits based on social evolution theory. I concur with their arguments. However, social evolution theory is more nuanced than currently presented in the paper, and makes additional predictions. For instance, it predicts that the relative shareability of secreted compounds varies in response to a number of factors, including the spatial structure of the environment and local cell density. With regard to surface-attached biofilms, it was shown that compound shareability is reduced in more viscous media and when cell density is low (van Gestel et al. 2014 ISME J; Weigert et al. 2017 Proc B). When translating these insights to the authors' study system, it would mean that resistant mutants resuming matrix production could experience some local benefits and increase in frequency under the above-mentioned conditions. I ask the authors to discuss these potential treatment limitations in the discussion.

2) Along the same lines, I would like to see a brief discussion on the Irie et al. 2017 mBio paper, where it was shown that matrix components, although being public goods, cannot be exploited in P. aeruginosa. This would mean that the logic of evolutionarily robust built up for the Salmonella system might not necessarily hold for other biofilm systems.

3) From the results (line 85, fig. 1f), it is unclear why fimbriae can be considered "public goods". They are attached to the cell, and so not shared in the classical sense (as diffusible public goods). I see how

they can affect the surrounding cells, but the social nature of fimbriae must be better explained. Furthermore, fig. 1f shows that there is complementation, but I'm not sure it shows shareability. A more direct test would be to harvest supernatent from producers and feed it to non-producers to see whether the colony phenotype is rescued. This should at least work for cellulose.

4) Figure 3. This figure makes an interesting point regarding drug concentration. A dose of 50 uM does not show any toxic effects on planktonic cells and yields evolutionary robust treatment results, while 75 uM shows some toxic effects and there are some patterns compatible with resistance evolution (e.g. increased cell no. in biofilm, fig. 3f). I have a number of questions related to this observation. (i) what would the authors predict to happen at even higher concentrations, e.g. 100 uM? (ii) if the evolutionary robustness is drug-concentration dependent, how could this be implemented in a host, where drug concentration at the target site is difficult to control? (iii) The finding matches those found for other anti-virulence compounds (e.g. gallium), where toxic effects kick in at higher concentrations. Could the authors' briefly discuss the problems that could arise in terms of evolutionary robustness in this context?

5) The authors should provide more information on the strains used for the "spread of resistance test" presented in Fig. 4. I agree that it is a conundrum to test whether resistant mutants can spread when no resistant mutants arise. That is perhaps why some of the previous studies have not performed such tests. So, I appreciate the authors' innovative approach. Still, this part is the weakest of the paper since the strains used in figure 4 are different from the regular wild type used. To make this part stronger, the reader needs to learn more about the background of these strains and how they differ from one another. Finally, I was wondering why the authors did not compete the "resistant" strain against their regular sensitive wild type? Would this not be the better comparison and better match the other parts of the paper?

Minor points:
- line 58: typo "gallium"

- line 90/91: Two times "to show" in the same sentence. Please revise.

- line 98: What are "problem Salmonella biofilms"? Please revise.

- fig. 1e: What is meant by "more structured"? Please explain.

- line 111: Not sure whether reference to unpublished data is allowed.

- line 220: Space is missing between "conventional" and "antimicrobial".

- figure 4a: it is strange that the anti-biofilm compound is only presented in the last figure of the paper. Could this panel be moved forward?

Signed: Rolf Kümmerli

Reviewer #2:
Remarks to the Author:
The authors put together a nice set of social evolution results on biofilm (EPS) production in

Salmonella (demonstrating indiv cost, collective benefit), and in agreement with existing studies on targeting cooperative pathogen traits they demonstrate that an 'anti-cooperation' compound is evolutionarily robust. In a key step that moves this study to the forefront of this research area – they isolate a (somewhat) resistant strain and demonstrate this strain is less competitive under drug selection – consistent with social evolution theory.

The paper is well written, the topic is important, however I have a few concerns that need to be addressed
–
At key points in the MS the work is positioned in the context of antibiotic resistance crisis and the critical need for novel therapeutics. However, at no point is there any discussion on the efficacy of this novel compound as a therapeutic. Does the compound work in a mouse or any animal model? Are the concentrations under study tolerated in a mammal?

The production of EPS is presented as a cooperative 'public good', imposing costs on the producers and benefits to neighbouring cells regardless of genotype. Figures 1 and 2 support these conclusions very nicely. However there's a big question over the generality of these results given a chunk of prior work demonstrating that EPS can be competitive trait allowing EPS+ lineages to out-compete EPS- lineages (https://www.pnas.org/content/104/3/876; https://www.pnas.org/content/108/34/14181, etc). The implication is that an EPS inhibitor in the (Vibrio) context of the studies above would select for inhibitor resistance due to direct competitive advantage of EPS production. That said, if it works in vivo for Salmonella then great! But the limits of application need to be clearly spelt out. These earlier studies suggest that the extent of cooperation (or conflict) across lineages depends on details of the strains under study and the environmental context.

Minor - the resistant strain results are important as I flagged above, however it needs to be clearly stated that these competition results use non-isogenic strains and therefore the menu of interaction mechanisms between these strains (beyond R versus S) is undefined.

Reviewer #3:
Remarks to the Author:
REVIEW NCOMMS-18-22067

The paper entitled " Inhibiting bacterial cooperation is an evolutionarily robust anti-biofilm strategy" by Dieltjens et al. focuses on the demonstration that a certain type of strategy to inhibit biofilms, here targeting EPS public goods, does not lead to evolution of resistance.

This type of study mixing information with clinical importance and concepts on evolution of bacteria is actually relatively rare.
There is clearly a dire need to rapidly identify strategies that both target biofilms, a well-known threat in medical environment, and are evolutionary resistant to avoid facing in few years the problems we are facing nowadays with multi-antibiotic resistance in bacteria.

There are 3 major claims in the present manuscript:
- the demonstration that EPS present in the biofilm matrix are public goods for Salmonella Typhimurium.
- the use of an EPS inhibitor, here a specific member of the class of 5-aryl-2-aminoimidazoles, against S. Typhimurium biofilms does not lead to evolution of resistance.

- additionally, the presence of this EPS inhibitor can also counter select resistant strains.
Conceptually the fact that EPS produced within biofilms can be considered public goods has been demonstrated in different species including B. subtilis (van Gestel J et al. 2014. Density of founder cells affects spatial pattern formation and cooperation in Bacillus subtilis biofilms. ISME J 8:2069–2079), V. cholerae (Drescher K et al. 2014. Solutions to the public goods dilemma in bacterial biofilms. Curr Biol 24:50–55) or P. aeruginosa (Irie Y et al. 2017. The Pseudomonas aeruginosa PSL polysaccharide is a social but no cheatable trait in biofilms. mBio 8:e00374-17). The formal demonstration that EPS of S. Typhimurium are public goods is thus not novel by itself but is a necessary information towards the development of a novel potential evolutionary resistant drug.
The demonstration that an EPS inhibitor does not lead to evolution of resistance is new as is the fact that this inhibitor can counter-select resistant strains.
The manuscript is elegantly written, very interesting and the experiments are generally well performed.

There are however some concerns in the manuscript that actually prevent me to be, for the moment, totally convinced by the study. The authors might be able to provide some explanations or perform some extra-experiments to remove any doubt.

Major concerns:
1- To me the major concern of this study is related to the use, along the whole story, of a csgD mutant instead of a double mutant of the genes coding the two EPS component cellulose and curli. In E. coli at least (and this should be check in the Salmonella literature or experimentally tackle), the CsgD protein is a global regulator that has been shown not only to regulate EPS component such as cellulose or curli production but also an important number of other genes including flagella genes (Dudin, O. et al (2014). Repression of flagellar genes in exponential phase by CsgD and CpxR, two crucial modulators of Escherichia coli biofilm formation. Journal of Bacteriology, 196(3), 707–715 or Ogasawara H et al. Role of the biofilm master regulator CsgD in cross-regulation between biofilm formation and flagellar synthesis. J Bacteriol. 2011;193(10):2587–2597, and may be in Salmonella as well Tan MS et al. Role of Fimbriae, Flagella and Cellulose on the Attachment of Salmonella Typhimurium ATCC 14028 to Plant Cell Wall Models. PLoS One. 2016;11(6):e0158311) or O-antigen genes (Gibson, D. L. et al. (2006). Salmonella produces an O-antigen capsule regulated by AgfD and important for environmental persistence. Journal of Bacteriology, 188(22), 7722–7730) that encode factors not known to be public goods and can strongly modulate biofilm formation ; also, RpoS stabilization is modulated by CsgD (Gualdi, L et al. (2007). Biofilm formation-gene expression relay system in Escherichia coli: modulation of sigmaS-dependent gene expression by the CsgD regulatory protein via sigmaS protein stabilization. Journal of Bacteriology, 189(22)) such that a csgD mutant has destabilized RpoS thus an affected response to stress in general including the one tested in the present manuscript. Also a csgD mutant might suffer from a fitness cost that is public good EPS independent.
- This to say that when using a csgD mutant in their experiments it is difficult to uncouple its role in regulating public goods such as curli and cellulose from its role in regulating other genes which modification of expression could impact fitness, biofilm formation and biofilm stress resistance.
- It is wrong to assimilate EPS production to csgD levels since the EPS of biofilm is not only made of curli and cellulose, and contains molecules which production does not depend on csgD.
- Crystal violet staining does not reflect only EPS but also stain the bacteria themselves, and staining of the EPS is not restricted to curli and cellulose.

While it will be unfair to ask for repeating all experiments with a double ΔcsgA_ΔbcsA mutant, I think it will be worse to verify some of the important claims of the paper with this double mutant strain. This strain has already been constructed in the ATCC 14028 background, please see Tan MS et al.

Role of Fimbriae, Flagella and Cellulose on the Attachment of Salmonella Typhimurium ATCC 14028 to Plant Cell Wall Models. PLoS One. 2016;11(6):e0158311).

2- In Figure 1f, the control with ΔcsgD rdar morphotype is missing. Rdar morphotype of the double mutant Δcurli_Δcellulose could also be added here. Using calcofluor staining on plates as well as in confocal biofilm observation could help directly looking at cellulose production or localization.

3- Line 148-149: "As expected, biofilm formation decreases with increasing proportion of the mutant (Supplementary Figure S3)." In Figure S3 is apparently described the total number of cells in the biofilm AND in the plankton above the biofilms. Thus how to be sure that the observed decreased is due to decrease in biofilm cells only and not to planktonic cells only or both?

4- Regarding the effect of the EPS inhibitor a missing control in the corresponding series of experiment is to verify that biofilm formation of the ΔcsgD or better the ΔcsgA-ΔbcsA double mutant is insensitive to the inhibitor.

5- Line 204-205: In Figure 3a, since the P value for the endpoint 0 μM vs 50 μM is equal to 0.0752, the authors cannot say that after evolution there is no change in EPS inhibition since their results suggest that at 50 μM the EPS inhibitor does not affect the end point evolved population. 75 μM however does (Figure 3e).

6- One important question is the mode of action of the EPS inhibitor. The authors indicated that it decreased the expression of csgD. Do the authors have more information on how this inhibitor is actually acting to reduce the expression of csgD. This is of particular importance since it can potentially help understanding why no resistant mutations can evolved to protect the cells from this inhibitor. Why the inhibitor starts to inhibit planktonic growth at 75 μM (Figure 3g)? Does it tell us about its mode of action?

7- The absence of evolution toward resistance against the EPS inhibitor at 50 μM is somehow not surprising since at this concentration the inhibitor actually do not kill or reduce the growth rate of the bacteria (Figure 3c), and no pressure is maintained to somehow force the bacteria to compensate their loss of biofilm formation capacity. It might be interesting to see what will happened if evolution is performed in presence of 75 μM of inhibitor where some growth inhibition is observed. Evolution at this higher concentration might allow to identify and isolate some mutated clones with increased resistance against the inhibitor, that for example have a reduced import of the inhibitor within the cells.

8- The work related to the competition between the sensitive and resistant strains is not totally convincing. I understand the necessity to use other strains if no real resistant mutant can be obtained in the strain ATCC 14028. However, here there is actually no information on the two chosen strains regarding their production of the two EPS compounds curli and cellulose, the public goods targeted in this manuscript. Referring to Figure 4a to state that "When untreated, EPS production of both strains is similar, although slightly higher for the R strain" (line 255-267) is wrong since number of cells in a biofilm does not always correlate with EPS production.

Some information is provided on the expression of csgD in biofilms formed by the two chosen strains but there is no formal proof about the regulation of these compounds by CsgD in these strains. In E. coli and Salmonella there is a high variability in the production of curli and cellulose in different strains that is reflected by the diversity of rdar morphotypes when inoculating strains collection. A minimum could be to show the rdar morphotype/calcofluor staining of these strains as compared to control strains including ATCC 14028. Some mutants (csgD, csgA, bcsA) could be constructed to see their behavior in front of the EPS inhibitor. Ideally the sensitivity of a double mutant ΔcsgA_ΔbcsA of the two strains should be tested to show that when deleting the two public goods encoding genes the two strains display the same absence of sensitivity. Also it seems to me that before competing the two strains in biofilm, they should also be competed in planktonic conditions as a control.

9- If we think about clinical application and we consider that a concentration around 50 μM of this inhibitor is necessary to maintain its effect while not allowing evolution of resistance it might be a bit tricky to find the best adapted treatment, depending of the biofilm-associated infections to treat, to sustain this concentration. In vitro, it might be possible to control the concentration of the inhibitor to

deliver; in vivo or in clinical situation this would be much more difficult. I think the authors should discuss these aspects.

10- While I agree that single strategies where resistance evolution never occurs would be more desirable than combination therapies, it seems to me that thinking that using a single EPS inhibitor in clinical situation of biofilm-related infection is unrealistic and actually dangerous if considering curative strategies. The reason is that dispersing biofilms without providing in addition an anti-microbial molecule that will kill biofilms dispersed cells will clearly conduct to treatment failure and high rate of mortality. As a consequence, these single therapies targeting EPS could only be applied for preventive strategies knowing that this type of strategies can be complicated to develop and to validate in clinics. The authors should discuss these aspects.

Minor concerns:

- Generally there is little M&M information that is given in the legend of supplementary figures. Some efforts should be made on this aspect.
- Is there a specific reason why the authors have chosen to use a ΔcsgB mutant for the minor curlin gene instead of a ΔcsgA mutant for the major curlin gene?
- csgD expression is bistable or heterogeneous in S. Typhymurium biofilms (Grantcharova et al. Bistable expression of csgD in biofilm development of Salmonella enterica Serovar Typhimurrium. J Bacteriol. 2010 Jan;192(2):456-66). How this information can be integrated in the reflection to target csgD-dependent EPS factors?
- Line 103-106: for the authors information, a Δcellulose mutant of S. Enteritidis has been previously shown to produce biofilms that are more susceptible to chlorine (see Solano, C. et al. (2002). Genetic analysis of Salmonella enteritidis biofilm formation: critical role of cellulose. Molecular Microbiology, 43(3), 793–808.
- Line 111 : « csgD is expressed during late-exponential phase (Van Puyvelde et al., unpublished data)", this is actually already described in ref 50.
- No statistics in Figure S3 and S4.

Typo errors:

- Line 34: established not estabilshed
- Line 58: gallium not galluim
- Line 220: a space is missing between conventional and antimicrobial.
- Line 327 of the M&M section: effect of DMSO on worm colonization or survival of infected worms?? Probably a left over from a previous version.

Detailed responses to the reviewers comments:

Reviewer #1 (Remarks to the Author):

The paper by Dieltjens et al. examines whether treatments inhibiting biofilm formation in bacterial pathogens is an evolutionarily robust strategy, where resistant mutants cannot spread in the population. Biofilm formation is based on the secretion of matrix components, which are shared as 'public good' at the level of the group. The authors argue that treatments inhibiting the production of such biofilm matrix components could be evolutionarily robust, because resistant phenotypes would involve the resumption of matrix production, which is costly to the individual cell, but generates benefits to everyone in the group, including the susceptible cells.

To test their hypotheses, the authors conducted a series of experiments with Salmonella typhimurium and the biofilm inhibitor 2-cyclopentenyl-5-(4-chlorophenyl)-2-aminoimidazole. They show that:

a) Matrix components are public goods that are costly to produce for the individual cell, but benefit the group as a whole (fig. 1).

b) Matrix non-producers can exploit matrix producers at all mixing ratios, and increase in frequency, thereby reducing biofilm formation and bringing cooperation to collapse (fig. 2).

c) Resistance against the anti-biofilm inhibitor does not evolve in in-vitro batch-culture experiments (fig. 3).

d) A resistant mutant cannot spread under treatment (fig. 4).

This is a nice and concise piece of work. Similar treatments and concepts have been applied to target cooperative siderophore secretion and quorum-sensing based communication in bacteria, but this is, to my knowledge, the first study that applies the social evolution concept to biofilm treatments. While the paper is very well written and the logic of the arguments are clear and easy to follow, I have a number of points I would like to see addressed:

Thank you for these positive and constructive comments. We agree that the main novelty is the application of the social evolution concept to biofilm treatments and the direct demonstration of outcompetition of a resistant strain. To address your remarks, in the revised manuscript and below we have (i) carefully discussed potential treatment limitations due to limited shareability of EPS under specific conditions, (ii) added new experiments to clarify the exploitability of the 2 main EPS types: curli fimbriae and cellulose; (iii) discussed concerns with regard to drug concentrations and (iv) added new experiments to further characterize the natural resistant and sensitive natural isolates.

1) The authors put forward strong arguments regarding the evolutionary robustness of treatments targeting social traits based on social evolution theory. I concur with their arguments. However, social evolution theory is more nuanced than currently presented in the paper, and makes additional predictions. For instance, it predicts that the relative shareability of secreted compounds varies in response to a number of factors, including the spatial structure of the environment and local cell density. With regard to surface-attached biofilms, it was shown that compound shareability is reduced in more viscous media and when cell density is low (van Gestel et al. 2014 ISME J; Weigert et al. 2017 Proc B). When translating these insights to the authors' study system, it would mean that resistant mutants resuming matrix

production could experience some local benefits and increase in frequency under the above-mentioned conditions. I ask the authors to discuss these potential treatment limitations in the discussion.

The shareability –and thus exploitability- of EPS and other public goods indeed depends on the population spatial structure and local cell density. We agree with the reviewer that this provides potential limitations to the treatment. However, one important point to note is that at the time a mutant EPS producer (resistant to an EPS inhibitor) first arises in a biofilm, it will very likely be surrounded by EPS non-producers in close proximity, meaning that it will initially experience a mixed genotype condition no matter what the population structure of other genotypes. We now discuss this and the potential treatment limitations in detail in a new section in the text (line 186-203).

2) Along the same lines, I would like to see a brief discussion on the Irie et al. 2017 mBio paper, where it was shown that matrix components, although being public goods, cannot be exploited in P. aeruginosa. This would mean that the logic of evolutionarily robust built up for the Salmonella system might not necessarily hold for other biofilm systems.

Certain cooperative EPS types - and also EPS types that specifically evolved to compete with non-producers - can indeed not be exploited. These EPS types are therefore not suitable as targets for our proposed strategy. We added a discussion and several references on this limitation in the text (line 186-203).

3) From the results (line 85, fig. 1f), it is unclear why fimbriae can be considered "public goods". They are attached to the cell, and so not shared in the classical sense (as diffusible public goods). I see how they can affect the surrounding cells, but the social nature of fimbriae must be better explained. Furthermore, fig. 1f shows that there is complementation, but I'm not sure it shows shareability. A more direct test would be to harvest supernatent from producers and feed it to non-producers to see whether the colony phenotype is rescued. This should at least work for cellulose.

This is an interesting point. Our experience with adding supernatant to biofilms or colonies in other species is that the resulting data are often inconclusive. We, therefore, decided to take a genetic approach to this question, which is possible for Salmonella where so much is known about the genetic bases of its EPS. Specifically, we assessed the relative contribution of curli fimbriae and cellulose to the observed fitness advantage of EPS non-producers in biofilms by competing the wild type against the ΔcsgA (no fimbriae) and ΔbcsA (no cellulose) mutant. This suggests that, in fact, it is the fimbriae that are most important for the fitness advantage of non-producers because a ΔcsgA single mutant outcompetes the wild type to a higher extent than the ΔbcsA single mutant (Figure S5d). Consistently, a ΔcsgAΔcsgB double mutant is able to outcompete the ΔbcsA mutant exploiting its curli fimbriae, but not the ΔcsgA single mutant (Figure S5d).

Like the reviewer, however, we think it is unlikely that the exploitation of curli fimbriae arises from a direct sharing of CsgA curlin subunits and polymerisation into fimbriae on the surface of the ΔcsgA mutant, because earlier studies have shown that the wild type strain (unlike a ΔcsgB mutant) is not a good donor of CsgA (Evans et al., 2014; Hammar et al., 1996). A more likely explanation is that curli non-producers attach to producers and increase their ability to attach, and remain attached, within a biofilm. Consistently, earlier work has shown that curli fimbriae are indispensable for both surface adhesion and cell aggregation (Jonas et al., 2007; Römling et al., 2000). Cellulose on the other hand was shown to be

less crucial for surface attachment and aggregation, although it strengthens the intercellular interactions (Zogaj et al.,2008; Römling et al., 2000). We have now clarified this model in the main text (Lines 173-185).

4) Figure 3. This figure makes an interesting point regarding drug concentration. A dose of 50 uM does not show any toxic effects on planktonic cells and yields evolutionary robust treatment results, while 75 uM shows some toxic effects and there are some patterns compatible with resistance evolution (e.g. increased cell no. in biofilm, fig. 3f). I have a number of questions related to this observation. (i) what would the authors predict to happen at even higher concentrations, e.g. 100 uM? (ii) if the evolutionary robustness is drug-concentration dependent, how could this be implemented in a host, where drug concentration at the target site is difficult to control? (iii) The finding matches those found for other anti-virulence compounds (e.g. gallium), where toxic effects kick in at higher concentrations. Could the authors' briefly discuss the problems that could arise in terms of evolutionary robustness in this context?

Our data indicate, rather strikingly, that although resistance against the toxic effect quickly develops at 75 µM, the antibiofilm activity is always preserved. Specifically, Fig 3e and h indicate that no changes in biomass and csgD inhibition occur due to selection. Moreover, cell number (Fig 3f) remains inhibited to an equal extent as after treatment with 50 µM inhibitor (Fig 3b) or when csgD is genetically turned off (Fig 1b). Our data therefore suggest that difficult control of drug concentration (too high dosing) is not a major concern for biofilm inhibition because only the toxic effects of excess drug are lost, while antibiofilm susceptibility is preserved.

5) The authors should provide more information on the strains used for the "spread of resistance test" presented in Fig. 4. I agree that it is a conundrum to test whether resistant mutants can spread when no resistant mutants arise. That is perhaps why some of the previous studies have not performed such tests. So, I appreciate the authors' innovative approach. Still, this part is the weakest of the paper since the strains used in figure 4 are different from the regular wild type used. To make this part stronger, the reader needs to learn more about the background of these strains and how they differ from one another. Finally, I was wondering why the authors did not compete the "resistant" strain against their regular sensitive wild type? Would this not be the better comparison and better match the other parts of the paper?

We now provide more information on the origin of both strains in the materials and methods section. The sensitive S strain (SGSC2227) is Salmonella enterica subsp. enterica serovar Paratyphi B (var. Java) str. CFSAN000529 S. isolated from sewage in Scotland in 1983, whereas the R strain (SGS3068) is Salmonella enterica subsp. diarizonae str. CFSAN000558 isolated from human in Oregon in 1987.

We have also added new experiments to further characterize the S and R natural isolates and their reliance on EPS components curli fimbriae and cellulose to form biofilms. We show that both strains have a rdar morphotype on CR-agar plates which indicates that they are producing cellulose and curli fimbriae (as was reported before for the R strain) (Fig S11) (White and Surette, 2006). We further constructed ΔcsgD, ΔbcsA, ΔcsgA and ΔbcsAΔcsgA mutants in the S strain and a ΔbcsA mutant in the R strain (we did not manage to construct the other mutants in the R strain because our phage transduction protocol was highly inefficient in this strain, but checked by sequencing that also this strain encodes csgD). All

mutants show the expected morphotypes on CR-agar plates further validating the role of curli fimbriae and cellulose and CsgD regulation in these strains (Fig S11). We next showed that mixing a ΔbcsA and ΔcsgA mutant of the S strain restores its rdar morphotype, confirming that curli and cellulose are shared in this strain. Also mixing ΔbcsA mutant of the R strain with the ΔcsgA mutant of the S strain restores the rdar morphotype indicating the EPS components are also shared between both strains. Given the earlier finding that exploitation of curli fimbriae appears to contribute most strongly to the relative fitness benefit of EPS non-producers, it is especially relevant that this experiment shows that curli fimbriae from the R strain (ΔbcsA mutant) are shared with the S strain (ΔcsgA mutant). Finally we validated that the EPS inhibitor targets csgD in the S strain, by showing that the residual biofilm formation of the ΔcsgD mutant of the S strain is insensitive to the inhibitor.

We did not directly compare the R strain with the regular sensitive wild type because both strains strongly differ in the level of biomass produced, even in the absence of inhibitor.

Minor points:
- line 58: typo "gallium" → *corrected*

- line 90/91: Two times "to show" in the same sentence. Please revise. → *revised*

- line 98: What are "problem Salmonella biofilms"? Please revise. → *revised*

- fig. 1e: What is meant by "more structured"? Please explain. → *The wild type appears to form clusters of cells, whereas the csgD mutant forms a thin layer of cells without clusters. We clarified this in the text.*

- line 111: Not sure whether reference to unpublished data is allowed. → *we referred to two earlier paper that also describe this finding.*

- line 220: Space is missing between "conventional" and "antimicrobial". → *corrected*

- figure 4a: it is strange that the anti-biofilm compound is only presented in the last figure of the paper. Could this panel be moved forward? → *We feel that including this panel in earlier figures would either affect the consistency or layout of the figures. However, should the editors be happy to make this panel into a new separate figure, we agree this would be a better option.*

Reviewer #2 (Remarks to the Author):

The authors put together a nice set of social evolution results on biofilm (EPS) production in Salmonella (demonstrating indiv cost, collective benefit), and in agreement with existing studies on targeting cooperative pathogen traits they demonstrate that an 'anti-cooperation' compound is evolutionarily robust. In a key step that moves this study to the forefront of this research area – they isolate a (somewhat) resistant strain and demonstrate this strain is less competitive under drug selection – consistent with social evolution theory.

The paper is well written, the topic is important, however I have a few concerns that need to be addressed

Thank you for this positive feedback. We agree that the main novelty of our work lies in the direct demonstration that a resistant strain is less competitive under drug selection than a sensitive strain, consistent with social evolution theory. To address your questions we have (i) clarified the clinical application potential of the selected EPS inhibitor, (ii) spelled out limits of application due to strain and condition dependency of EPS exploitation and (iii) explicitly stated in the text that the R and S strain are non-isogenic strains of which the menu of interactions –beyond EPS- is undefined.

–

At key points in the MS the work is positioned in the context of antibiotic resistance crisis and the critical need for novel therapeutics. However, at no point is there any discussion on the efficacy of this novel compound as a therapeutic. Does the compound work in a mouse or any animal model? Are the concentrations under study tolerated in a mammal?

Salmonella biofilms cause major problems in industrial settings as contaminants, as well as in clinical settings. The experiments we present, therefore, do model an important potential application of the inhibitor in a manner that will help to alleviate the rise of resistant strains.

In the revised text, we better substantiated the application potential of the 2-aminoimidazole based EPS inhibitors by discussing and referring to our previous application oriented work (Line 226-237). Specifically, 2-aminoimidazoles can be coated to surfaces – both covalently (Peeters et al., 2019) or in a slow-release matrix (Claes et al., 2017)– to prevent biofilm formation in food industry or on medical surfaces. However, as the reviewer rightly notes, there is also the potential for the inhibitors to be used in clinical contexts to treat infections without promoting resistance evolution. Here we obviously need to be more cautious based on in-vitro data but the potential for clinical application is supported by our recent reports showing that 2-aminoimidazoles can inhibit biofilm formation in vivo in a subcutaneous model in rats (Peeters et al.2019), have low cytotoxicity against different mammalian cell types (tumor cell lines and bone cells) and do not affect the survival of Caenorhabditis elegans (Steenackers et al., 2014, Peeters et al., 2016), a small nematode that is widely used for toxicity testing and is considered to have high predictive value for toxicity in mammals. We now discuss this potential application of the inhibitors in the discussion.

The production of EPS is presented as a cooperative 'public good', imposing costs on the producers and benefits to neighbouring cells regardless of genotype. Figures 1 and 2 support these conclusions very nicely. However there's a big question over the generality of these results given a chunk of prior work

demonstrating that EPS can be competitive trait allowing EPS+ lineages to out-compete EPS- lineages (https://www.pnas.org/content/104/3/876; https://www.pnas.org/content/108/34/14181, etc). The implication is that an EPS inhibitor in the (Vibrio) context of the studies above would select for inhibitor resistance due to direct competitive advantage of EPS production. That said, if it works in vivo for Salmonella then great! But the limits of application need to be clearly spelt out. These earlier studies suggest that the extent of cooperation (or conflict) across lineages depends on details of the strains under study and the environmental context.

We agree with this comment and now carefully discuss these limitations in the revised manuscript (Line186-203).

Minor - the resistant strain results are important as I flagged above, however it needs to be clearly stated that these competition results use non-isongenic strains and therefore the menu of interaction mechanisms between these strains (beyond R versus S) is undefined.

We have explicitly spelled out this limitation in the revised text (Line 329-331).

Reviewer #3 (Remarks to the Author):

The paper entitled " Inhibiting bacterial cooperation is an evolutionarily robust anti-biofilm strategy" by Dieltjens et al. focuses on the demonstration that a certain type of strategy to inhibit biofilms, here targeting EPS public goods, does not lead to evolution of resistance.

This type of study mixing information with clinical importance and concepts on evolution of bacteria is actually relatively rare.
There is clearly a dire need to rapidly identify strategies that both target biofilms, a well-known threat in medical environment, and are evolutionary resistant to avoid facing in few years the problems we are facing nowadays with multi-antibiotic resistance in bacteria.

There are 3 major claims in the present manuscript:
- the demonstration that EPS present in the biofilm matrix are public goods for Salmonella Typhimurium.
- the use of an EPS inhibitor, here a specific member of the class of 5-aryl-2-aminoimidazoles, against S. Typhimurium biofilms does not lead to evolution of resistance.
- additionally, the presence of this EPS inhibitor can also counter select resistant strains.

Conceptually the fact that EPS produced within biofilms can be considered public goods has been demonstrated in different species including B. subtilis (van Gestel J et al. 2014. Density of founder cells affects spatial pattern formation and cooperation in Bacillus subtilis biofilms. ISME J 8:2069–2079), V. cholerae (Drescher K et al. 2014. Solutions to the public goods dilemma in bacterial biofilms. Curr Biol 24:50–55) or P. aeruginosa (Irie Y et al. 2017. The Pseudomonas aeruginosa PSL polysaccharide is a social but no cheatable trait in biofilms. mBio 8:e00374-17). The formal demonstration that EPS of S. Typhimurium are public goods is thus not novel by itself but is a necessary information towards the development of a novel potential evolutionary resistant drug.
The demonstration that an EPS inhibitor does not lead to evolution of resistance is new as is the fact that this inhibitor can counter-select resistant strains.
The manuscript is elegantly written, very interesting and the experiments are generally well performed.

There are however some concerns in the manuscript that actually prevent me to be, for the moment, totally convinced by the study. The authors might be able to provide some explanations or perform some extra-experiments to remove any doubt.

Thank you for this summary that recognises the general relevance of our work. To address the main concerns we have (i) constructed a ΔbcsAΔcsgA double mutant and repeated key experiments to validate that the relative fitness advantage of the ΔcsgD mutant is specifically related to reduced production of cellulose and curli fimbriae, (ii) added morphotypes of ΔcsgD and ΔbcsAΔcsgA mutants as a control, (iii) shown that ΔcsgD mutant is insensitive to the inhibitor confirming that it targets csgD, (iv) clarified the concentration dependency of activity of and resistance against the inhibitor, (v) further characterized the sensitive and resistant natural isolates in rdar assays (both with wild type and EPS mutants of these strains), (vi) validated that the EPS inhibitor also targets csgD in the sensitive natural strain, (vii) added more M&M information on supplementary figures and (viii) replaced all data on ΔcsgB mutant with data on ΔcsgA mutant.

Major concerns:

1- To me the major concern of this study is related to the use, along the whole story, of a csgD mutant instead of a double mutant of the genes coding the two EPS component cellulose and curli. In E. coli at least (and this should be check in the Salmonella literature or experimentally tackle), the CsgD protein is a global regulator that has been shown not only to regulate EPS component such as cellulose or curli production but also an important number of other genes including flagella genes (Dudin, O. et al (2014). Repression of flagellar genes in exponential phase by CsgD and CpxR, two crucial modulators of Escherichia coli biofilm formation. Journal of Bacteriology, 196(3), 707–715 or Ogasawara H et al. Role of the biofilm master regulator CsgD in cross-regulation between biofilm formation and flagellar synthesis. J Bacteriol. 2011;193(10):2587–2597, and may be in Salmonella as well Tan MS et al. Role of Fimbriae, Flagella and Cellulose on the Attachment of Salmonella Typhimurium ATCC 14028 to Plant Cell Wall Models. PLoS One. 2016;11(6):e0158311) or O-antigen genes (Gibson, D. L. et al. (2006). Salmonella produces an O-antigen capsule regulated by AgfD and important for environmental persistence. Journal of Bacteriology, 188(22), 7722–7730) that encode factors not known to be public goods and can strongly modulate biofilm formation ; also, RpoS stabilization is modulated by CsgD (Gualdi, L et al. (2007). Biofilm formation-gene expression relay system in Escherichia coli: modulation of sigmaS-dependent gene expression by the CsgD regulatory protein via sigmaS protein stabilization. Journal of Bacteriology, 189(22)) such that a csgD mutant has destabilized RpoS thus an affected response to stress in general including the one tested in the present manuscript. Also a csgD mutant might suffer from a fitness cost that is public good EPS independent.

- This to say that when using a csgD mutant in their experiments it is difficult to uncouple its role in regulating public goods such as curli and cellulose from its role in regulating other genes which modification of expression could impact fitness, biofilm formation and biofilm stress resistance.
- It is wrong to assimilate EPS production to csgD levels since the EPS of biofilm is not only made of curli and cellulose, and contains molecules which production does not depend on csgD.
- Crystal violet staining does not reflect only EPS but also stain the bacteria themselves, and staining of the EPS is not restricted to curli and cellulose.

While it will be unfair to ask for repeating all experiments with a double ΔcsgA_ΔbcsA mutant, I think it will be worse to verify some of the important claims of the paper with this double mutant strain. This

strain has already been constructed in the ATCC 14028 background, please see Tan MS et al. Role of Fimbriae, Flagella and Cellulose on the Attachment of Salmonella Typhimurium ATCC 14028 to Plant Cell Wall Models. PLoS One. 2016;11(6):e0158311).

To the best of our knowledge, regulation of O-antigen capsule (Gibson et al., 2006) and BapA protein (Latasa et al., 2005) by CsgD has so far only been shown in S. Enteritidis and not in S. Typhimurium (Zakikhany K et al., 2010)) and regulation of flagella by CsgD only in E. coli (Dudin et al., 2014) although there are data suggesting that CsgD also regulates flagella in Salmonella (Zakikhany et al., 2010). Nevertheless, we very much agree that it is good to validate that the fitness advantage of the csgD mutant is specifically related to reduced production of cellulose and curli fimbriae. We therefore compared the ΔcsgD mutant with a ΔbcsAΔcsgA double mutant in a number of key experiments. The double mutant has a 'saw' (smooth and white) morphotype (Figure 1f) like the ΔcsgD mutant, and shows a similar reduction in biomass in the 48 h biofilm assay (Figure S5a) and increase in growth rate in well-mixed liquid culture as the ΔcsgD mutant (Figure S5b). Consistently, the double mutant outcompetes the wild type to a similar extent as the ΔcsgD mutant in the biofilim assay across a wide range of initial frequencies (10 % to 90 % of mutant) (Figure S5c). Exploitation of curli fimbriae appears to contribute most strongly to the relative fitness advantage of EPS non-producers given the finding that a ΔcsgA single mutant outcompetes the wildtype to a higher extent than a ΔbcsA single mutant (Figure S5d). Consistently, the double mutant is able to outcompete the ΔbcsA mutant exploiting its curli fimbriae, but not the ΔcsgA single mutant (Figure S5d). This information has been added in lines 166-185.

2- In Figure 1f, the control with ΔcsgD rdar morphotype is missing. Rdar morphotype of the double mutant Δcurli_Δcellulose could also be added here. Using calcofluor staining on plates as well as in confocal biofilm observation could help directly looking at cellulose production or localization.

We added the saw morphotypes (the ΔcsgD and ΔbcsAΔcsgA mutant) as controls in Fig 1f.

3- Line 148-149: "As expected, biofilm formation decreases with increasing proportion of the mutant (Supplementary Figure S3)." In Figure S3 is apparently described the total number of cells in the biofilm AND in the plankton above the biofilms. Thus how to be sure that the observed decreased is due to decrease in biofilm cells only and not to planktonic cells only or both?

Our apologies The legend of this graph by mistake indicated that it shows the total number of cells in the biofilm plus plankton, while it actually shows the total number of cells of wild type plus ΔcsgD mutant in the biofilm only. We have corrected the legend.

4- Regarding the effect of the EPS inhibitor a missing control in the corresponding series of experiment is to verify that biofilm formation of the ΔcsgD or better the ΔcsgA-ΔbcsA double mutant is insensitive to the inhibitor.

We have added this control at line 242-243 and in Fig S6.

5- Line 204-205: In Figure 3a, since the P value for the endpoint 0 μM vs 50 μM is equal to 0.0752, the authors cannot say that after evolution there is no change in EPS inhibition since their results suggest that at 50 μM the EPS inhibitor does not affect the end point evolved population. 75 μM however does (Figure 3e).

We have added additional technical replicates of the measurements for the 3 parallel evolved populations to increase the statistical power. With these additional repeats, the endpoint 50 µM is significantly lower than the endpoint 0 µM. Moreover, the endpoint 50 µM is not significantly higher than the parental 50 µM (also in the original graph) further indicating that resistance did not develop.

6- One important question is the mode of action of the EPS inhibitor. The authors indicated that it decreased the expression of csgD. Do the authors have more information on how this inhibitor is actually acting to reduce the expression of csgD. This is of particular importance since it can potentially help understanding why no resistant mutations can evolved to protect the cells from this inhibitor. Why the inhibitor starts to inhibit planktonic growth at 75 µM (Figure 3g)? Does it tell us about its mode of action?

This is indeed an interesting question and details on the mode of action of the EPS inhibitor are subject of ongoing investigation in our lab. Unfortunately, at this point we do not have enough data to reach conclusions.

7- The absence of evolution toward resistance against the EPS inhibitor at 50 µM is somehow not surprising since at this concentration the inhibitor actually do not kill or reduce the growth rate of the bacteria (Figure 3c), and no pressure is maintained to somehow force the bacteria to compensate their loss of biofilm formation capacity.

Respectfully, we don't agree. In our serial passage evolution experiments we only passaged biofilm cells attached to the surface, which generates a strong selection pressure for biofilm-associated traits that allow cells to attach. Evidence that this assay does indeed select for biofilm formation is supported by control experiments performed in the absence of the inhibitor, where we observe an evolutionary response of increased biofilm formation via increased attachment (Figure S7). As a result, an inhibitor of biofilm formation is indeed expected to exert strong selection for bacteria to become resistant and be able to develop biofilm in the presence of the inhibitor. We now make this critical point clearer in the manuscript.

It might be interesting to see what will happened if evolution is performed in presence of 75 µM of inhibitor where some growth inhibition is observed. Evolution at this higher concentration might allow to identify and isolate some mutated clones with increased resistance against the inhibitor, that for example have a reduced import of the inhibitor within the cells.

This experiment was actually in the original manuscript (bottom part of Fig 3 (e-h). Strikingly, we found that at this concentration resistance against toxic effects rapidly occurs, but that the inhibition of EPS remains preserved (Line 269-287). That is, we see that natural selection for toxicity and biofilm formation show divergent responses even in the same experiment.

8- The work related to the competition between the sensitive and resistant strains is not totally convincing. I understand the necessity to use other strains if no real resistant mutant can be obtained in the strain ATCC 14028. However, here there is actually no information on the two chosen strains regarding their production of the two EPS compounds curli and cellulose, the public goods targeted in this manuscript. Referring to Figure 4a to state that "When untreated, EPS production of both strains is similar, although slightly higher for the R strain" (line 255-267) is wrong since number of cells in a biofilm does not always correlate with EPS production.
Some information is provided on the expression of csgD in biofilms formed by the two chosen strains but there is no formal proof about the regulation of these compounds by CsgD in these strains. In E. coli and Salmonella there is a high variability in the production of curli and cellulose in different strains that is

reflected by the diversity of rdar morphotypes when inoculating strains collection. A minimum could be to show the rdar morphotype/calcofluor staining of these strains as compared to control strains including ATCC 14028. Some mutants (csgD, csgA, bcsA) could be constructed to see their behavior in front of the EPS inhibitor. Ideally the sensitivity of a double mutant ∆csgA_∆bcsA of the two strains should be tested to show that when deleting the two public goods encoding genes the two strains display the same absence of sensitivity. Also it seems to me that before competing the two strains in biofilm, they should also be competed in planktonic conditions as a control.

These are good questions. As also described above (in response to reviewer 1), we have now added additional information on the origin of the strains in the materials and methods section. We also added experiments to further characterize the S and R natural isolates and their reliance on EPS components curli fimbriae and cellulose to form biofilms. We showed that both strains have a rdar morphotype on CR-agar plates which indicates that they are producing cellulose and curli fimbriae (as was reported before for the R strain) (Fig S11). We constructed ∆csgD, ∆bcsA, ∆csgA and ∆bcsA∆csgA mutants in the S strain and a ∆bcsA mutant in the R strain (we did not manage to construct the other mutants in the R strain because our phage transduction protocol was highly inefficient in this strain, but checked by sequencing that also this strain encodes csgD). All mutants show the expected morphotypes an CR-agar plates further validating the role of curli fimbriae and cellulose and CsgD regulation in these strains (Fig S11). We next showed that mixing a ∆bcsA and ∆csgA mutant of the S strain restores its rdar morphotype, confirming that curli and cellulose are shared in this strain. Also mixing ∆bcsA mutant of the R strain with the ∆csgA mutant of the S strain restores the rdar morphotype indicating the EPS components are also shared between both strains. Given the above finding that exploitation of curli fimbriae appears to contribute most strongly to the relative fitness benefit of EPS non-producers, it is especially relevant that this experiment shows that curli fimbriae from the R strain (∆bcsA mutant) are shared with the S strain (∆csgA mutant). Finally we validated that the EPS inhibitor targets csgD in the S strain, by showing that the residual biofilm formation of the ∆csgD mutant of the S strain is insensitive to the inhibitor.

With regard to differences in amount of EPS production of both strains we do not only refer to cell number, but also to biomass staining with crystal violet (Fig 4b) and expression levels of csgD (Fig. 4c), the same metrics used earlier as a proxy to quantify EPS upon exposure to the EPS inhibitor or in the ∆csgD mutant.

We also competed the R and S strain in planktonic conditions as a control with and without inhibitor (Fig S15d). Under these conditions, the two strains behave as expected according to our model for their behaviour under biofilm conditions. Specifically, the sensitive strain which makes less EPS without the inhibitor outcompetes the resistant strain in absence of inhibitor. And this effect becomes more pronounced in the presence of inhibitor which reduces EPS more in sensitive than the resistant strain (Figure 4e). In competition then, the outcome is the same as in biofilms but importantly in monoculture, the sensitive strain reaches a higher cell density than the resistant strain, which differs from biofilm conditions where it is the resistant strain which makes more EPS that does better. These results are all exactly as expected because EPS production in plankton is costly but not beneficial, such that the strain that makes the least EPS is expected to reach the highest cell number (a similar pattern is observed when competing csgD mutant with the WT in plankton).

9- If we think about clinical application and we consider that a concentration around 50 µM of this

inhibitor is necessary to maintain its effect while not allowing evolution of resistance it might be a bit tricky to find the best adapted treatment, depending of the biofilm-associated infections to treat, to sustain this concentration. In vitro, it might be possible to control the concentration of the inhibitor to deliver; in vivo or in clinical situation this would be much more difficult. I think the authors should discuss these aspects.

As discussed above, our data indicate, rather strikingly, that although resistance against the toxic effect quickly develops at 75 µM, the antibiofilm activity is still preserved. Our data therefore suggest that difficult control of drug concentration (too high dosing) is not a major concern since only the toxic effects of excess drug will be lost, whereas antibiofilm susceptibility appears to be preserved.

10- While I agree that single strategies where resistance evolution never occurs would be more desirable than combination therapies, it seems to me that thinking that using a single EPS inhibitor in clinical situation of biofilm-related infection is unrealistic and actually dangerous if considering curative strategies. The reason is that dispersing biofilms without providing in addition an anti-microbial molecule that will kill biofilms dispersed cells will clearly conduct to treatment failure and high rate of mortality. As a consequence, these single therapies targeting EPS could only be applied for preventive strategies knowing that this type of strategies can be complicated to develop and to validate in clinics. The authors should discuss these aspects.

The proposed inhibitors prevent EPS production and biofilm formation but are not expected to break down existing biofilms, at least not at the shorter term. We therefore rather envision applications where the EPS inhibitors are applied in a preventive setting, i.e. (i) by compound coatings in order to prevent contamination of industrial or medical surfaces or (ii) as a preventive drug to inhibit Salmonella colonization in the gut when the risk of exposure to Salmonella is high, as is currently done with probiotics and vaccines. We have added a discussion section to clarify how we see the practical application of the compounds (line 226-237) and we referred to our earlier work on (i) development of covalent and slow-release coatings of the EPS inhibitors to surfaces (Peeters et al., 2019; Claes et al., 2017), (ii) low cytotoxicity of the inhibitors against eukaryotic cells and C. elegans (Steenackers et al., 2014; Peeters et al., 2016) and (iii) in vivo preventive activity in a subcutaneous rat model (Peeters et al., 2019).

Minor concerns:
- Generally there is little M&M information that is given in the legend of supplementary figures. Some efforts should be made on this aspect.
We added more M&M information on supplementary figures both in the general M&M section and in the figure legends.

- Is there a specific reason why the authors have chosen to use a ΔcsgB mutant for the minor curlin gene instead of a ΔcsgA mutant for the major curlin gene?
We have replaced all the data for the ΔcsgB mutant with data for a ΔcsgA mutant.

- csgD expression is bistable or heterogeneous in S. Typhymurium biofilms (Grantcharova et al. Bistable expression of csgD in biofilm development of Salmonella enterica Serovar Typhimurrium. J Bacteriol. 2010 Jan;192(2):456-66). How this information can be integrated in the reflection to target csgD-dependent EPS factors?

This is an interesting question. Bistability in expression of cooperative genes has the potential to slow down the speed by which cheaters rise in frequency. This has been shown earlier for genes encoding virulence factors in S. Typhimurium that promote an inflammatory response that allows Salmonella to outcompete commensal microbiota. Expression of the virulence genes is bistable leading to a slow-growing subpopulation that expresses virulence genes and a fast-growing population that does not express the virulence genes. The presence of the fast-growing non-expression was shown to slow down exploitation by fast-growing cheater strains that do not produce the virulence factors at all, but reap the benefits of the inflammatory response (Diard M. et al. 2013 – Nature 494(7437):353-6). Similarly, bistability in csgD expression might slow down EPS exploitation in Salmonella. However, given we do see EPS exploitation, in our experiments at least we can be confident that any such effect is insufficient to overcome cheating.

- Line 103-106: for the authors information, a Δcellulose mutant of S. Enteritidis has been previously shown to produce biofilms that are more susceptible to chlorine (see Solano, C. et al. (2002). Genetic analysis of Salmonella enteritidis biofilm formation: critical role of cellulose. Molecular Microbiology, 43(3), 793–808.

We have added this reference at this position in the text.

- Line 111 : « csgD is expressed during late-exponential phase (Van Puyvelde et al., unpublished data)", this is actually already described in ref 50.

We referred to this reference and reference 54 instead.

- No statistics in Figure S3 and S4.

Figure 2a shows that the ΔcsgD mutant has a significantly higher biofilm accumulation in coculture than the wild type over the 48h period of biofilm formation. Figure S4 suggests that the biofilm accumulation of the ΔcsgD mutant is higher during each stage of biofilm formation, however adding statistics indicated that the differences are not statistically significant. We might therefore have overemphasized the data. We now downtoned this statement in the text and removed the graph in the new version of the paper. Most importantly though, the time course indicates that the ΔcsgD mutant gains in frequency throughout the 48h, with the largest proportion of change (53,7 %) occurring during growth in the biofilm after attachment (12-48 h) (Figure 2d).

We have added statistics to S3.

Typo errors → *all corrected*

- Line 34: established not estabilshed
- Line 58: gallium not galluim
- Line 220: a space is missing between conventional and antimicrobial.
- Line 327 of the M&M section: effect of DMSO on worm colonization or survival of infected worms?? Probably a left over from a previous version.

Reviewers' Comments:

Reviewer #1:
Remarks to the Author:
The authors have adequately addressed my comments. The revised version reads very well and represents a convincing evidence of how targeting social virulence traits can be effective and evolution proof. I'm looking forward to the next stage where this therapy is tested in hosts.

I have only one very minor point:

On line 168, please specify why a double mutant was used to validate the previous results. From the text it is not clear what this mutant is and what it can or (cannot) do. Please clarify.

Reviewer #2:
Remarks to the Author:
The authors have responded constructively to my critiques.

Reviewer #3:
Remarks to the Author:
The authors made great efforts to follow reviewer's recommendation in term of clarification of some results or methods, of additional discussion and of novel experiments. In its current form the manuscript is clearly improved.

Detailed responses to the reviewers comments:

Reviewer #1 (Remarks to the Author):

The authors have adequately addressed my comments. The revised version reads very well and represents a convincing evidence of how targeting social virulence traits can be effective and evolution proof. I'm looking forward to the next stage where this therapy is tested in hosts.

I have only one very minor point:

On line 168, please specify why a double mutant was used to validate the previous results. From the text it is not clear what this mutant is and what it can or (cannot) do. Please clarify.

Thank you for this positive feedback.

We now indicate that this mutant lacks the cellulose synthase catalytic subunit BcsA and the major curlin subunit CsgA and can therefore not produce curli and cellulose (line 121-122). In addition, we clarify that we use this mutant because in related strains and species, CsgD has been shown to regulate a number of additional loci, next to those involved in production of the EPS components cellulose and curli. It is therefore important to validate that the relative fitness advantage of the ΔcsgD mutant in biofilms is specifically related to reduced production of cellulose and curli fimbriae (line 165-169).

Reviewer #2 (Remarks to the Author):

The authors have responded constructively to my critiques.

Thank you for this positive feedback.

Reviewer #3 (Remarks to the Author):

The authors made great efforts to follow reviewer's recommendation in term of clarification of some results or methods, of additional discussion and of novel experiments. In its current form the manuscript is clearly improved.

Thank you for this constructive feedback.
